# The Trials and Tribulations of Structure Assisted Design of K_Ca_ Channel Activators

**DOI:** 10.3389/fphar.2019.00972

**Published:** 2019-09-20

**Authors:** Heesung Shim, Brandon M. Brown, Latika Singh, Vikrant Singh, James C. Fettinger, Vladimir Yarov-Yarovoy, Heike Wulff

**Affiliations:** ^1^Department of Pharmacology, School of Medicine, University of California, Davis, Davis, CA, United States; ^2^Department of Chemistry, University of California, Davis, Davis, CA, United States; ^3^Department of Physiology and Membrane Biology, School of Medicine, University of California, Davis, Davis, CA, United States

**Keywords:** calcium-activated potassium channels, K_Ca_3.1, K_Ca_2.2, calmodulin binding domain, SKA-111, Rosetta, K_Ca_ activators

## Abstract

Calcium-activated K^+^ channels constitute attractive targets for the treatment of neurological and cardiovascular diseases. To explain why certain 2-aminobenzothiazole/oxazole-type K_Ca_ activators (SKAs) are K_Ca_3.1 selective we previously generated homology models of the C-terminal calmodulin-binding domain (CaM-BD) of K_Ca_3.1 and K_Ca_2.3 in complex with CaM using Rosetta modeling software. We here attempted to employ this atomistic level understanding of K_Ca_ activator binding to switch selectivity around and design K_Ca_2.2 selective activators as potential anticonvulsants. In this structure-based drug design approach we used RosettaLigand docking and carefully compared the binding poses of various SKA compounds in the K_Ca_2.2 and K_Ca_3.1 CaM-BD/CaM interface pocket. Based on differences between residues in the K_Ca_2.2 and K_Ca_.3.1 models we virtually designed 168 new SKA compounds. The compounds that were predicted to be both potent and K_Ca_2.2 selective were synthesized, and their activity and selectivity tested by manual or automated electrophysiology. However, we failed to identify any K_Ca_2.2 selective compounds. Based on the full-length K_Ca_3.1 structure it was recently demonstrated that the C-terminal crystal dimer was an artefact and suggested that the “real” binding pocket for the K_Ca_ activators is located at the S4-S5 linker. We here confirmed this structural hypothesis through mutagenesis and now offer a new, corrected binding site model for the SKA-type K_Ca_ channel activators. SKA-111 (5-methylnaphtho[1,2-*d*]thiazol-2-amine) is binding in the interface between the CaM N-lobe and the S4-S5 linker where it makes van der Waals contacts with S181 and L185 in the S_45_A helix of K_Ca_3.1.

## Introduction

Small- and intermediate-conductance calcium-activated potassium channels (K_Ca_) are voltage independent and are gated by the binding of calcium to calmodulin, which functions as their calcium-sensing beta subunit (Xia et al., 1998; Fanger et al., 1999). There are four members in the small- and intermediate-conductance K_Ca_ channel subfamily, the small-conductance K_Ca_2.1, K_Ca_2.2, and K_Ca_2.3, collectively known as SK channels, and the intermediate-conductance K_Ca_3.1, also known as IK (Kaczmarek et al., 2017). These tetrameric membrane proteins consist of four six-transmembrane domains with their N- and C-termini positioned intracellularly. Calmodulin (CaM) is constitutively bound with its C-lobe to the calmodulin binding domain (CaM-BD) in the C-terminus of each K_Ca_ channel subunit and opens the channel when the N-lobe binds calcium following increases in intracellular calcium in the proximity of the channel (Xia et al., 1998; Lee and MacKinnon, 2018). K_Ca_2/3 channels are differentially expressed in the human body with K_Ca_2 channels primarily expressed in, but not limited to, the CNS and K_Ca_3.1 primarily found in peripheral tissues, lymphocytes and red blood cells (Adelman et al., 2012; Wulff and Kohler, 2013). K_Ca_2 channels mediate after hyperpolarization and regulate firing frequency in neurons (Adelman et al., 2012). K_Ca_3.1, on the other hand, is responsible for generating the driving force for calcium influx in immune cells and contributes to the regulation of vascular tone *via* the vascular endothelium (Wulff and Kohler, 2013). These expression patterns make both K_Ca_2 and K_Ca_3.1 channels attractive pharmacological targets (Wulff and Zhorov, 2008). Specifically, activation of K_Ca_2 channels has been proposed for the treatment of diseases characterized by increased neuronal excitability, like ataxia (Shakkottai et al., 2011; Kasumu et al., 2012) and epilepsy, whereas activation of K_Ca_3.1 has been proposed as a treatment for hypertension (Wulff and Kohler, 2013) and as a possible way to pharmacologically enhance anti-tumor T cell responses (Chandy and Norton, 2016). The idea behind the later hypothesis is that by enhancing K^+^ efflux and thus lowering intracellular K^+^ it might be possible to reset the “ionic checkpoint” and boost anti-tumor T cell functions in tumor infiltrating T cells, which have been shown to have increased intracellular K^+^ concentrations suppressing their ability to activate (Eil et al., 2016). In support of this exciting therapeutic postulate, it has recently been demonstrated that pharmacological K_Ca_3.1 activation can restore the ability of cancer patient derived CD8^+^ T cells to chemotax (Chimote et al., 2018).

Identifying potent and selective K_Ca_ channel activators has been challenging. The first generation, which includes 1-EBIO, NS309 (Strobaek et al., 2004), and SKA-31 (Sankaranarayanan et al., 2009), are relatively unselective and only display a 5–10-fold selectivity for K_Ca_3.1 over K_Ca_2 channels (Christophersen and Wulff, 2015). This lack of selectivity has led to CNS related side effects when trying to use K_Ca_3.1 activation as a new, endothelial targeted antihypertensive approach (Radtke et al., 2013). The second generation of K_Ca_ channel activators, as exemplified by SKA-121 and SKA-111, are 40–100-fold selective for K_Ca_3.1 and were efficacious in lowering blood pressure in normotensive and hypertensive mice while avoiding K_Ca_2 channel mediated side effects on the CNS and on heart rate because of the improvement in selectivity (Coleman et al., 2014). For the development of our first and second generation K_Ca_ activators (the “SKA” compounds), we used a classical medicinal chemistry approach with no structural input during the structure activity optimization (Sankaranarayanan et al., 2009; Coleman et al., 2014). However, more recently, following the publication (Zhang et al., 2013) of the crystal structure of the K_Ca_2.2 CaM-BD in complex with CaM and containing NS309 (pdb: 4J9Z), we generated homology models of the K_Ca_3.1 and K_Ca_2.3 CaM-BD in complex with CaM using the Rosetta molecular modeling suite and RosettaLigand for compound docking (Brown et al., 2017). Combining structural modeling and site-directed mutagenesis we determined that S372 in K_Ca_3.1 (or the corresponding S632 in K_Ca_2.3) is crucial for the activity of all 2-aminobenzothiazole/oxazole-type K_Ca _activators, which are further stabilized by an extensive hydrogen bond network including E295, N300, R362 in K_Ca_3.1 and M51 and E54 in calmodulin. Based on our findings we suggested that R362, a residue which is at the center of this network in the K_Ca_3.1 but not the K_Ca_2.3 models, is responsible for the 5–10-fold selectivity of these compounds for K_Ca_3.1 over K_Ca_2.3 (Brown et al., 2017).

In the current work we set out to use our atomistic level understanding of K_Ca_ activator binding to develop a third generation of K_Ca_ channel activators; this time with selectivity for K_Ca_2.2 over K_Ca_3.1. In this structure-based drug design approach we again used Rosetta ligand docking and carefully compared the binding poses of 2-aminobenzothiazole/oxazole-type K_Ca_ activators in the K_Ca_2.2 and K_Ca_3.1 CaM-BD/CaM interface pocket. Based on differences between residues in the K_Ca_2.2 and K_Ca_.3.1 models we manually designed 168 new virtual SKA compounds and tried to predict whether the compounds would show selectivity for K_Ca_2.2 based on the computational docking models. The most promising compounds were synthesized, and their potency and selectivity for K_Ca_2.2, and K_Ca_3.1 tested by manual or automated electrophysiology. However, despite all these efforts, we failed to identify any K_Ca_2.2 selective compounds and recently learned from the full-length cryo-EM structure of K_Ca_3.1 published by the MacKinnon group (Lee and MacKinnon, 2018), that the C-terminal crystal was an artefact. Based on the full-length structure it was suggested that the “real” binding pocket for the K_Ca_ activators is located between the S4-S5 linker and the CaM N-lobe (Lee and MacKinnon, 2018). We here confirmed this structural hypothesis through mutagenesis and now offer a new, corrected binding site model for the SKA-type K_Ca_ channel activators. We hope that our findings provide a cautionary tale for the field in warning against some of the pitfalls in structure-based drug design.

## Material and Methods

### Molecular Modeling

We previously described the generation of models (Brown et al., 2017) of the K_Ca_3.1 and K_Ca_2.3 C-terminal CaM-binding domain in complex with CaM with Rosetta computational modeling software (Rohl et al., 2004; Bender et al., 2016; Alford et al., 2017) using the x-ray structure of the K_Ca_2.2 channel CaM-binding domain in complex with CaM and NS309 (Zhang et al., 2013) (pdb id: 4J9Z) as a template. We also previously provided a detailed description (Brown et al., 2017) of the procedure for ligand docking using the RosettaLigand docking application (Meiler and Baker, 2006; Davis and Baker, 2009; Bender et al., 2016) in the Rosetta program suite, version 3.7. Briefly, for this study, ligand conformers of variously substituted benzothiazoles/oxazoles were generated using Open Eye OMEGA software version 2.5.1.4 (OpenEye Scientific Software, Santa Fe, NM; http://www.eyesopen.com) (Hawkins et al., 2010; Hawkins and Nicholls, 2012; OEChem v OpenEye Scientific Software, Inc.), randomly placed within the binding pocket and then taken through the three stages of the RosettaLigand modeling which progresses from low-resolution conformational sampling and scoring to full atom optimization using Rosetta’s all-atom energy function. A total of 10,000 models were generated for each virtual compound, the top 1,000 models with the lowest total energy score were selected, and the top 10 models with the lowest binding energy were identified, manually inspected for ligand/channel interactions and convergence between the K_Ca_2.2 and the K_Ca_3.1 model compared. A model was considered converged if the top 10 models overlaid.

Cryo-EM structures of the full length K_Ca_3.1 channel (Lee and MacKinnon, 2018) in two open states and one closed state (pdb id: 6CNN, 6CNO, 6CNM) were refined using the Rosetta cryo-EM refinement protocol (Wang et al., 2016) (Cryo-EM density map, Rosetta version 3.8) and the models with the lowest energy scores were chosen for docking of SKA-111. All molecular graphics of ligand, K_Ca_ channel C-terminal CaM-binding domain in complex with CaM, or full length K_Ca_3.1 channels were rendered using the UCSF Chimera software (Resource for Biocomputing, Visualization, and Informatics, San Francisco, CA).

Potential druggable sites in the Rosetta refined K_Ca_3.1 open state-1 structure were identified using the SiteMap function in Glide (Schrödinger, LLC, New York, NY, 2018). SiteMap uses a site localization method based on interaction energies between the protein and grid probes, which is analogous to the Goodford’s GRID algorithm. Sites were kept if they comprised at least 50 site points. A restrictive hydrophobicity definition and a standard grid (1.0 Å) were used for identifying potential binding pockets.

Protein Data Bank (pdb) format files of the Rosetta models of K_Ca_3.1 open state 1 and open state 2 with SKA-111 docked in the interface between S_45_A helix and the CaM N-lobe are provided in the Data Supplement^1^; pdb files of all other models are available upon request.

### Molecular Biology

The cloning of human K_Ca_3.1 (#AF033021) has been reported in the late 1990s (Logsdon et al., 1997). The gene was subcloned in-frame downstream to green fluorescent protein in the pEGFP-C1 expression vector (CLONTECH) (Wulff et al., 2001). All clones were verified by sequencing. Mutations were introduced using QuikChange site directed mutagenesis kit (Stratagene, La Jolla, CA) and were verified by fluorescence sequencing. For amino acid numbering for K_Ca_3.1 we used the gene: *Homo sapiens* potassium channel, calcium activated intermediate/small conductance subfamily N alpha, member 4 (*KCNN4*). NCBI Reference Sequence: NP_002241.1.

### Electrophysiology

All experiments were performed in either the inside-out or the whole-cell configuration of the patch-clamp technique on either transiently transfected CHO cells or Human Embryonic Kidney (HEK) cell lines stably expressing hK_Ca_3.1 or hK_Ca_2.2 (Sankaranarayanan et al., 2009). All cells were cultured in Dulbecco’s modified Eagle’s medium supplemented with 10% fetal calf serum. Wild-type (WT) and mutant hK_Ca_3.1 channel constructs were transfected using FuGENE 6 transfection reagent (Promega, Madison, WI) in OptiMEM reduced-serum medium (Life Technologies, Benicia, CA) for manual patch-clamp experiments or *via* electroporation (Nucleofector 2b and Lonza Amaxa Cell Line Nucleofector Kit T) according to the manufacturer’s instructions for automated electrophysiology.

Cells transfected using FuGENE 6 were cultured in six-well plates for 24–48 h and then detached by TrypLE Express (Gibco, Grand Island, NY) and plated on coverslips for 30 min to 1 h for whole-cell recordings. For inside-out recordings, cells were plated 2–3 h before the experiments to attach them more firmly. Coverslips were placed in a 15 ml recording chamber mounted on an inverted microscope (Olympus XI-70 equipped with a pE-300Lite LED UV light source and filters; Olympus, Tokyo, Japan), and only clearly green fluorescent cells were patch-clamped. CHO cells transfected *via* electroporation were cultured in T75 flasks until 70% confluency and lifted with TrypLE, spun down, and resuspended. 1X10^6^ cells were used per transfection with 1 µg of plasmid DNA. The setting for the Nucleofector 2b was, cell type: CHO, high efficiency. Cells were afterwards cultured in a T25 flask for 24 h before they were lifted for electrophysiology.

For manual whole-cell experiments, the extracellular solution contained 160 mM NaCl, 4.5 mM KCl, 2 mM CaCl_2_, 1 mM MgCl_2_, and 10 mM HEPES (pH 7.4, 300 mOsm). Solutions on the intracellular side contained 154 mM KCl, 10 mM HEPES, 10 mM EGTA, 1.75 mM MgCl_2_, and 5.9 mM CaCl_2_ for a calculated free Ca^2+^ concentration of 250 nM (pH 7.2, 290 mOsm). For inside-out experiments in symmetrical K^+^, the extracellular solution contained 154 mM KCl, 10 mM HEPES, 1 mM MgCl_2_, and 2 mM CaCl_2_ (pH 7.4, 300 mOsm). Intracellular solutions contained 154 mM KCl, 10 mM HEPES, 10 mM EGTA, 1.75 mM MgCl_2_, and varying amounts of CaCl_2_ for calculated free Ca^2+^ concentrations of 0.05, 0.1, 0.25, 0.5, 1, 10, and 30 mM (pH 7.2, 280–300 mOsm). Free Ca^2+^ concentrations were calculated using the July 3, 2009 online version of MaxChelator (https://somapp.ucdmc.ucdavis.edu/pharmacology/bers/maxchelator/webmaxc/webmaxcS.htm) assuming a temperature of 25°C, a pH of 7.2, and an ionic strength of 160 mM. Patch pipettes were pulled from soda lime glass (micro-hematocrit tubes; Kimble Chase, Rochester, NY) and had resistances of 1.5–3 MΩ when submerged. Experiments were controlled with a HEKA EPC-10 amplifier and Patchmaster software (HEKA, Lambrecht/Pfalz, Germany). In whole-cell experiments, cells were clamped to a holding potential of -80 mV, and K_Ca_ currents were elicited by 200-ms voltage ramps from -120 to +40 mV applied every 10 s. For the inside-out experiments, cells were clamped to a holding potential of -80 mV, macro-patches were pulled and K_Ca_ currents were elicited by 200-ms voltage ramps from -80 to +80 mV applied every 5 s. Solutions with different free Ca^2+^ concentrations were then perfused in rapid successions to avoid run down. Each Ca^2+^ concentration was normalized to 10 µM of free Ca^2+^ by perfusing 10 µM Ca^2+^ before and after each Ca^2+^ concentration. If the two 10 µM values differed more than 10% from each other, the data point was excluded from the Ca^2+^ concentration response curve.

The procedure for performing automated whole-cell K_Ca_ channel recordings on a QPatch-16 automated electrophysiology platform (Sophion Biosciences) was previously described in detail by our laboratory (Jenkins et al., 2013). For the current study we used the solutions described above with disposable 16-channel planar patch chip plates (QPlates; patch hole diameter 1 mm, resistance 2.00–0.02MΩ, Sophion Biosciences, Woburn, MA). Cell positioning and sealing parameters were set as follows: positioning pressure -70 mbar, resistance increase for success 750%, minimum seal resistance 0.1 GΩ, holding potential -0 mV, holding pressure -20 mbar. To avoid rejection of cells with large K_Ca_3.1 currents, the minimum seal resistance for whole-cell requirement was lowered to 0.001 GΩ. Access was obtained with the following sequence: 1) suction pulses in 29 mbar increments from -250 mbar to -453 mbar; 2) a suction ramp of an amplitude of -450 mbar; 3) -400 mV voltage zaps of 1 ms duration. Following establishment of the whole-cell configuration, cells were held at -80 mV and K_Ca_ currents elicited by a voltage protocol that held at -80 mV for 20 ms, stepped to -120 mV for 20 ms, ramped from -120 to + 40 mV in 200 ms, and then stepped back to -120 mV for 20 ms. This pulse protocol was applied every 10 s. K_Ca_ activator dilutions were prepared freshly (within 5 min of the initiation of the QPatch) with Ringer’s solution from 10 mM stock solutions in dry DMSO. Final DMSO concentrations never exceeded 1%. Glass vial inserts (to avoid adsorption) were filled with 400 µL of compound solution and placed into the insert base plate for use in the QPatch assay. Exemplary QPatch raw current traces and plots of slope conductance versus time are shown in **Supplementary Figure 3**. For each compound we typically used 12 consecutive liquid periods Break-in, 3 saline additions to stabilize the current, 2 additions of test compound at 10 µM, 2 saline washes, 2 additions of SKA-31 at 10 µM, followed by 2 more saline washes. In each liquid period 10 pulse protocols were run.

Data analysis, fitting, and plotting were performed with IGOR-Pro (Wavemetrics, Lake Oswego, OR) or Origin 9 (OriginLab Corporation, Northampton, MA).

### Calcium Sensitivity Testing

When screening mutants, we always first assessed whether mutations had altered Ca^2+^ sensitivity in a two-step process. First, mutant channels were patched with an intracellular solution containing 10 µM of free Ca^2+^. If the mutant did not display currents with amplitudes in the nA range under these conditions, we then tested whether the channel’s control current (at 250 nM free Ca^2+^) could be increased 5–10-fold in the presence of 100 µM EBIO (this fold increase level is common in the wild type channel). If the mutant did not meet either threshold it was deemed to have altered Ca^2+^ sensitivity and was excluded from subsequent experiments investigating sensitivity to SKA-111. All mutants shown in **Figure 7** or mentioned in the text exhibited current densities at +40 mV that were comparable to the WT channel and produced currents that were large enough to perform experiments. WT: 19.5 ± 23.4 pA/pF; T212F-V272F: 12.4 ± 10.4 pA/pF; S181A: 11.6 ± 16.2 pA/pF; A184F: 7.2 ± 5.4 pA/pF; L185A: 6.9 ± 6.5 pA/pF; S181A-L185A: 8.8 ± 5.5 pA/pF; S372R: 13.8 ± 12.5 pA/pF (n = 5).

### Chemistry

#### Commercially Available Compounds

4-Biphenyl-4-yl-thiazol-2-ylamine (SKA-232, CAS 2834-79-9) was purchased from Sigma–Aldrich (St. Louis, MO). Bis-4,4’-(2-amino-4-thiazolyl)biphenyl (SKA-255, CAS 438233-93-3) was purchased from Oakwood Products, INC (West Columbia, SC). Identity and purity (<95%) of commercial compounds was confirmed by ^1^H NMR before using compounds for electrophysiological testing.

#### Chemical Synthesis

Compounds that were not commercially available were synthesized in our laboratory according to the general methods described below. Compounds reported previously were characterized by melting point, proton nuclear magnetic resonance (^1^H NMR) and 13 carbon (^13^C) NMR. All NMR spectra were recorded on an 800 MHz Bruker Avance III spectrometer. Data are reported as follows: chemical shift (δ), multiplicity, integration, coupling constant (Hz). Signals are designated as follows: s (singlet), d (doublet), dd (doublet of doublets), ddd (doublet of doublet of doublets), dt (doublet of triplets), t (triplet), quint (quintet), m (multiplet). New chemical entities were additionally characterized by high-resolution mass spectrometry (HRMS). For compounds (SKA-218, SKA-339, SKA-340, SKA-343 and SKA-347) where it was difficult to unambiguously confirm the structure based on ^1^H and ^13^C NMR, we grew crystals and subjected them to X-ray analysis, which allowed us to see the exact position of the substituents in the compounds. All compounds used for electrophysiological experiments were at least 95–98% pure based on NMR and/or mass spectrometry.

##### General Method I. Preparation of nitro-substituted 2-metylnaphtho[1,2-d]thiazoles or 1-substituted 4-nitronaphthalenes

One milliliter of HNO_3_ was added slowly to 1 g of 2-methyl-β-naphthothiazole or 1-substituted naphthalene in a round bottom flask at room temperature without solvent. The reaction mixture was stirred for 1 h and then 1.5 ml of H_2_SO_4 _(95%) was added slowly. The mixture was stirred at room temperature for 30 min until completion of the reaction was indicated by the disappearance of starting material on thin-layer chromatography (TLC). Then the pH of the reaction mixture was adjusted to between 7 and 7.5 with 4N NaOH, the mixture extracted with ethyl acetate and the organic phase washed with water and brine. The extract was dried with anhydrous sodium sulfate and solvent was evaporated under reduced pressure. The crude product was purified *via* flash-chromatography (petroleum ether: ethyl acetate, 8:2).

##### General Method II. Preparation of amino-substituted 2-metylnaphtho[1,2-d]thiazoles or 1-amino 4-substituted naphthalenes

One gram of the previously prepared nitro-substituted 2-metylnaphtho[1,2-*d*]thiazole or 1-substituted 4-nitronaphthalene was dissolved in 30 mL of 95% ethanol and 5% water, 20 mg of palladium charcoal was added and then 2 mL of hydrazine were dropped in slowly. After the addition was completed, the reaction mixture was stirred at 80°C for 12 h and the progress of the reaction was monitored by TLC with dichloromethane. After completion, the reaction mixture was filtered to remove the palladium charcoal and concentrated to dryness under vacuum. The residue was dissolved in ethyl acetate and was washed with water and brine. The organic phase was dried with anhydrous sodium sulfate and the solvent was removed under vacuum. The crude product was purified by flash-chromatography with ethyl acetate/petroleum ether (3:7 v/v) as eluent to give the product.

##### General Method III. Preparation of methyl-substituted acetonaphthones

Acetylchloride (750 μl, 10 mmol) and AlCl_3_ (1.33 g, 10 mmol) were added sequentially to 20 ml of chloroform at room temperature. Methyl substituted naphthalenes (1 ml, 7 mmol) in 5 ml of chloroform were then added, the reaction mixture was stirred for 2 h, and the progress of the reaction was monitored by TLC. The reaction mixture was quenched with sodium bicarbonate solution and washed with water and brine. Solvent was removed under reduced pressure to give the crude product. The crude product was purified by flash–chromatography using ethyl acetate/petroleum ether (2:8 v/v).

##### General Method IV. Preparation of substituted 2-bromo-1-(methyl substituted naphthalen-1-yl)ethan-1-one or 2-bromo-1-(methyl substituted cyclohex-1-yl)ethan-1-one

Appropriately substituted acetophenones were dissolved in chloroform (20 ml) at room temperature and the reaction mixture was stirred. Liquid bromine (1.2–1.5 equivalent) in chloroform (5 ml) was then added drop-wise. The progress of the reaction was monitored by TLC. The reaction mixture was quenched with a saturated aqueous solution of sodium hydrogen carbonate and the pH was adjusted to between 7 and 7.5. The organic phase was washed with water and brine and the solvent was removed under vacuum. The crude residue was purified *via* flash chromatography (petroleum ether: ethyl acetate, 7:3).

##### General Method V. Preparation of substituted 4-phenylthiazole or 4-naphthalenyl thiazoles

Substituted 2-bromo-1-(methyl substituted naphthalen-1-yl)ethan-1-one or 2-bromo-1-(methyl substituted cyclohex-1-yl)ethan-1-one was added to a solution of a substituted thiourea in 20 ml of absolute ethanol. The mixture was refluxed for 2 h. After completion of the reaction, the ethanol was evaporated under vacuum. The dried reaction mixture was dissolved in dichloromethane and neutralized with saturated NaHCO_3_. The organic phase was washed with water and brine, and then dried with anhydrous Na_2_SO_4_. Finally, the solvent was evaporated under vacuum. The crude product was reconstituted in a methanol, treated with charcoal and recrystallized.

##### General Method VI. Preparation of heterocyclic 1-substituted 4-nitronaphthalenes

1-Fluoro-4-nitronaphthalene and a secondary or heterocyclic amine (molar ratio 1:3) were dissolved in 10 mL of DMF at 90°C. Potassium carbonate (3 equivalents) was then added. The reaction mixture was stirred and monitored by TLC. After completion, the reaction mixture was washed with brine several times to remove the DMF and extracted with ethyl acetate. Solvent was evaporated to give the crude product. The crude product was purified by flash-chromatography using ethyl acetate/petroleum ether (2:8 v/v).

##### General Method VII. Preparation of substituted 2-aminonaphthothiazoles

Liquid bromine (150 μL, 3 mmol) and KSCN (485 mg, 5 mmol) were added to a solution of the 1-amino 4-substituted naphthalenes (3 mmol) in 10 ml of acetic acid and the reaction mixture was stirred at room temperature for 30 min. The reaction was quenched by adding 4N NaOH. The reaction mixture was then washed with water and brine and extracted with ethyl acetate. Solvent was evaporated to give the crude product, which was purified by flash-chromatography using ethyl acetate/petroleum ether (3:7 v/v).

##### General Method VIII. Preparation of N-substituted naphtho[1,2-d]thiazol-2-amine

1-Naphthylamine 500 mg (3.5 mmol) was dissolved in 25 mL chloroform and 0.3 mL of trimethylamine before adding substituted isothiocyanate (3.8 mmol). The resulting mixture was refluxed at 65°C for 48 h. Solvent was then evaporated, and the resulting solid was washed with diethyl ether to obtain *N,N’*-disubstituted thiourea. The *N,N’*-disubstituted thiourea (2.2 mmol) obtained in the previous step was suspended in 25 mL chloroform, and a solution of liquid bromine in chloroform (1 eq.) added over a period of 1 h, after which sodium thiosulfate (aq.) was added to the reaction mixture. The reaction mixture was washed with 4N NaOH (aq.) and the organic layer was dried over anhydrous sodium sulfate and evaporated under reduced pressure.

**2-Methylnaphtho[1,2-*d*]thiazole-5-carbonitrile (SKA-126).** To a solution of 5-bromo-2-methylnaphtho[1,2-*d*]thiazole (SKA-132, 280 mg, 1 mmol) in a mixture of DMF (3 mL) and water (200 uL), kept under nitrogen, were added copper (I) cyanide (CuCN, 121 mg, 1.33 mmol), Pd_2_(dba)_3_ (Tris (dibenzylideneacetone) dipalladium (0), 70 mg) and 1,1′-ferrocenediyl-bis(diphenylphosphine) (dppf, 80 mg). The resulting solution was heated at 110°C for 75 h, after which the mixture was filtered and diluted with 10 mL water and was extracted using chloroform (3X10 mL). The organic layer was separated, dried over anhydrous sodium sulfate and evaporated under reduced pressure. Chromatographic purification of the crude product on silica gel using ethyl acetate and hexane (10:90) furnished 120 mg (50%) of the required product as off white solid and 85 mg starting material: m.p = 139–140°C. ^1^H NMR (800 MHz, CDCl_3_): δ = 8.85–8.82 (m, 1H), 8.34–8.31 (m, 2H), 7.80–7.74 (m, 2H), 2.99 (s, 3H). ^13^C NMR (201 MHz, CDCl_3_): δ = 170.8, 152.7, 130.7, 130.6, 128.5, 128.30, 128.0, 126.5, 125.6, 124.7, 117.9, 107.3, 20.6. HRMS (ESI): m/z calculated for C_13_H_8_N_2_S (M+H)^+^: 225.0485 found: 225.0485.

**2-Aminonaphtho[1,2-*d*]oxazole-5-sulfonic acid (SKA-128).** To a solution of 4-amino-3-hydroxynaphthalene-1-sulfonic acid (1.0 g, 4.18 mmol) and 3.0 mL Hunig’s base in 50 mL (9:1) ethanol:water was added 850 mg of cyanogen bromide. The resulting mix was stirred at room temperature for 4 d. After completion of the reaction the solvent was evaporated, and the residue was dissolved in water. It was acidified by adding 1N HCl. The precipitate thus obtained was filtered, washed with acetone, and dried to render 800 mg (81%) of pink-brown solid: m.p = 295°C (Decomposes).^1^H NMR (800 MHz, DMSO-*d*_6_ & Et_3_N): δ = 8.92 (dt, *J* = 8.6, 0.9 Hz, 1H), 8.20 – 8.16 (m, 1H), 8.07 (s, 1H), 7.63 (s, 2H), 7.56 (ddd, *J* = 8.1, 6.7, 1.2 Hz, 1H), 7.50 (ddd, *J* = 8.3, 6.7, 1.5 Hz, 1H). ^13^C NMR (201 MHz, DMSO-*d*_6 _& Et_3_N): δ = 163.6, 141.8, 139.3, 136.9, 128.3, 126.8, 125.3, 124.2, 124.1, 121.5, 108.9. HRMS (ESI): m/z calculated for C_11_H_8_N_2_O_4_S (M+H)^+^: 265.0278 found: 265.0284.

**5-Chloro-2-methylnaphtho[1,2-*d*]thiazole (SKA-130).** 2-Methyl-β-naphthothiazole (400 mg, 2 mmol), *N*-chlorosuccinamide (266 mg, 2 mmol), NaCl (117 mg, 2 mmol) and P-TSOH (344 mg, 2 mmol) were dissolved in 30 ml of acetonitrile. The reaction mixture was stirred at 40°C for 2 h, and the progress of the reaction was monitored by TLC using an ethyl acetate:petroleum ether mixture (3:7). After completion, the reaction mixture was filtered and washed with water and acetonitrile. The residue was recrystallized from methanol as light yellow crystals (100 mg, 21%); m.p. = 103°C. ^1^H NMR (800 MHz, CDCl_3_): δ = 8.81 (dd, *J* = 8.2, 1.3 Hz, 1H), 8.37 (d, *J* = 8.3 Hz, 1H), 7.99 (s, 1H), 7.74 (ddd, *J* = 8.2, 6.8, 1.3 Hz, 1H), 7.70 (ddd, *J* = 8.3, 6.8, 1.4 Hz, 1H), 2.96 (s, 3H). ^13^C NMR (201 MHz, CDCl_3_),: δ = 166.3, 148.6, 131.3, 129.0, 129.0, 128.7, 127.6, 126.8, 124.9, 124.2, 118.9, 20.1. HRMS (ESI): m/z calculated for C_12_H_8_ClNS (M+H)^+^: 243.0138; found: 243.0135.

**5-Bromo-2-methylnaphtho[1,2-*d*]thiazole (SKA-132).** To a solution of 2-methylnaphtho[1,2-*d*]thiazole (1.0 g, 5 mmol) in 25 mL chloroform was added a solution of molecular bromine in chloroform (I eq) over a period of 2 min and the resulting solution was stirred for 30 min at room temperature after which sodium thiosulfate (aq.) was added. This was followed by washing with 4N NaOH (aq.). The organic layer was dried over anhydrous sodium sulfate and evaporated under reduced pressure to obtain 1202 mg (85%) The product was isolated as white solid; m.p = 98–99°C (CAS 125427-01-2).^1^H NMR (800 MHz, CDCl_3_): δ = 8.78 (dd, *J* = 8.1, 1.4 Hz, 1H), 8.33–8.30 (m, 1H), 8.16 (s, 1H), 7.70 (ddd, *J* = 8.1, 6.8, 1.3 Hz, 1H), 7.67 (ddd, *J* = 8.3, 6.9, 1.4 Hz, 1H), 2.93 (s, 3H). ^13^C NMR (201 MHz, CDCl_3_): δ = 166.5, 149.4, 132.0, 130.2, 129.0, 127.8, 127.7, 127.2, 124.4, 122.6, 119.6, 20.3.

**5-Bromo-2-methylnaphtho[1,2-*d*]oxazole (SKA-133).** SKA-133 was prepared from 2-methylnaphtho[1,2-*d*]oxazole (1.0 g, 5.45 mmol) following the method described above for the synthesis of SKA-132. The product was isolated as a white solid (90%); m.p = 101–102°C (CAS 1838658-03-9). ^1^H NMR (800 MHz, CDCl_3_): δ = 8.45 (dd, *J* = 8.2, 1.3 Hz, 1H), 8.33 (dd, *J* = 8.5, 1.1 Hz, 1H), 7.99 (s, 1H), 7.68 (ddd, *J* = 8.1, 6.9, 1.1 Hz, 1H), 7.63 (ddd, *J* = 8.3, 6.9, 1.3 Hz, 1H), 2.73 (s, 3H). ^13^C NMR (201 MHz, CDCl_3_): δ = 163.4, 147.6, 136.7, 129.3, 128.1, 127.8, 126.7, 126.6, 122.5, 118.9, 115.1, 14.8.

**Naphtho[2,3-*d*]oxazol-2-amine (SKA-134).** SKA-134 was prepared from 3-aminonaphthalen-2-ol and cyanogen bromide following the method described for the synthesis of SKA-128. The product was isolated as a white solid (97%); m.p = 247–248°C (CAS 1820618-76-5). ^1^H NMR (800 MHz, DMSO-*d*_6_): δ = 7.93–7.87 (m, 2H), 7.80 (d, *J* = 0.8 Hz, 2H), 7.76 (s, 3H), 7.61 (d, *J* = 0.9 Hz, 2H), 7.39 (dddd, *J* = 26.3, 8.3, 6.8, 1.5 Hz, 1H). ^13^C NMR (201 MHz, DMSO-*d*_6_): δ = 164.3, 148.5, 144.3, 131.6, 129.1, 127.9, 127.5, 124.5, 123.7, 110.8, 104.3.

**2-Methylnaphtho[1,2-*d*]oxazole-5-carbonitrile (SKA-135).** SKA-135 was prepared from 5-bromo-2-methylnaphtho[1,2-*d*]oxazole (SKA-133) and CuCN following the method described for the synthesis of SKA-126. The product was isolated as a brownish crystal (42%); m.p. = 145–146°C (CAS 60111-00-4). ^1^H NMR (800 MHz, CDCl_3_): δ = 8.50–8.46 (m, 1H), 8.31 (dt, *J* = 8.2, 0.9 Hz, 1H), 8.05 (s, 1H), 7.74 (ddd, *J* = 8.2, 6.9, 1.3 Hz, 1H), 7.71 (ddd, *J* = 8.3, 6.9, 1.4 Hz, 1H), 2.78 (s, 3H). ^13^C NMR (201 MHz, CDCl_3_): δ = 166.4, 146.2, 141.3, 130.5, 128.5, 127.8, 126.0, 125.9, 122.8, 117.9, 117.3, 106.4, 14.9.

***N*-Phenylnaphtho[1,2-*d*]thiazol-2-amine (SKA-146).** SKA-146 was prepared from 1-naphthylamine and phenylisothiocyanate according to General Method VIII. The product was isolated as a white solid (320 mg, 42%);****m.p = 140–141°C (CAS 21431-44-7). ^1^H NMR (800 MHz, DMSO-*d*_6_): δ = 10.67 (s, 1H), 8.58 (dt, *J* = 8.1, 1.0 Hz, 1H), 8.02 (dd, *J* = 8.1, 1.1 Hz, 1H), 7.99 – 7.94 (m, 3H), 7.75 (d, *J* = 8.5 Hz, 1H), 7.67 (ddd, *J* = 8.1, 6.8, 1.2 Hz, 1H), 7.58 (ddd, *J* = 8.1, 6.8, 1.3 Hz, 1H), 7.49 – 7.44 (m, 2H), 7.09 (tt, *J* = 7.3, 1.1 Hz, 1H). ^13^C NMR (201 MHz, DMSO-*d*_6_): δ = 162.3, 147.3, 140.8, 131.7, 129.1, 128.0, 126.2, 126.1, 125.4, 124.7, 123.4, 122.2, 121.9, 119.1, 117.5.

***N*-Methylnaphtho[1,2-*d*]thiazol-2-amine (SKA-158).** SKA-158 was prepared from1-naphthylamine and methylisothiocyanate according to General Method VIII. The product was isolated as a white solid (42%); m.p = 187–188°C (CAS 876484-23-0).^1^H NMR (800 MHz, DMSO-*d*_6_): δ = 8.45 (dd, *J* = 8.2, 1.2 Hz, 1H), 8.13 (d, *J* = 4.8 Hz, 1H), 7.97 – 7.84 (m, 2H), 7.60 (d, *J* = 8.5 Hz, 1H), 7.57 (ddd, *J* = 8.2, 6.7, 1.3 Hz, 1H), 7.51 (ddd, *J* = 8.1, 6.7, 1.3 Hz, 1H), 3.07 (d, *J* = 4.5 Hz, 3H). ^13^C NMR (201 MHz, DMSO-*d*_6_): δ = 168.1, 147.9, 131.6, 127.9, 125.8, 125.5, 125.1, 124.3, 123.5, 120.5, 119.3, 30.7.

***N*-Cyclohexylnaphtho[1,2-*d*]thiazol-2-amine (SKA-165).** 1-Naphthyl isothiocyanate (2 g, 10.8 mmol) was added to 20 mL of ethanol and the mixture was stirred and refluxed at 80°C. Then, 1.24 mL (10.8 mmol) of cyclohyxylaminenaphtho[1,2-*d*]thiazol-2-ylamine was added and the mixture refluxed for 2 h. The progress of the reaction was monitored by TLC. After completion of the reaction ethanol was removed under vacuum, the residue extracted with ethyl acetate and the organic phase washed with water followed by brine. The organic layer was dried with anhydrous Na_2_SO_4_ and the solvent was evaporated. 700 mg (2.46 mmol) of the intermediate product, 1-cyclohexyl-3-(naphthalen-1-yl)thiourea, was dissolved in 20 mL of chloroform. Then 131.1 uL (2.46 mmol) of liquid bromine was added dropwise. After completion the reaction was quenched by adding 4N NaOH, the resulting mixture was washed with water and brine and extracted with ethyl acetate. Finally, the solvent was evaporated under vacuum. The crude product was reconstituted in a methanol, treated with charcoal and recrystallized. The product was isolated as a brownish crystal. (460 mg, 17%); m.p = 115–116°C (CAS 1368045-48-0).^1^H NMR (800 MHz, DMSO-d_6_): δ = 8.38 (d, *J* = 8.2 Hz, 1H), 8.10 (d, *J* = 7.4 Hz, 1H), 7.89 (d, *J* = 8.0 Hz, 1H), 7.79 (d, *J* = 8.5 Hz, 1H), 7.56 - 7.50 (m, 2H), 7.46 (t, *J* = 7.4 Hz, 1H), 3.74 (s, 1H), 2.12 - 1.96 (m, 2H), 1.76 (dt, *J* = 13.1, 4.0 Hz, 2H), 1.61 (dt, *J* = 13.3, 4.0 Hz, 1H), 1.36 (dddd, *J* = 37.2, 15.2, 12.0, 5.8 Hz, 4H), 1.22 (dtd, *J* = 15.5, 11.8, 9.5, 6.0 Hz, 1H).^13^CNMR(201MHz, DMSO-*d*_6_): δ = 166.8, 148.3, 132.1, 128.3, 125.9, 125.52, 124.5, 123.9, 120.9, 119.7, 79.6, 53.7, 32.7, 25.7, 24.9.

**2-Methylnaphtho[1,2-*d*]thiazol-6-amine (SKA-172).** SKA-172 was prepared from 2-methyl-6-nitronaphtho[1,2-*d*]thiazole (SKA-215) in 2 steps according to General methods I and II. The resulting residue was purified by flash-chromatography with ethyl acetate/petroleum ether (3:7 v/v) as eluent to give SKA-172 as reddish crystals (40 mg, 19%); m.p. = 148–150°C. ^1^H NMR (800 MHz, CDCl_3_): *δ* = 8.08 (dd, *J* = 8.9, 0.8 Hz, 1H), 7.94 (d, *J* = 8.9 Hz, 1H), 7.87 (dt, J = 8.1, 1.0 Hz, 1H), 7.38 (t, J = 7.8 Hz, 1H), 6.81 (dd, *J* = 7.6, 1.1 Hz, 1H), 5.89 (s, 2H), 2.91 (s, 3H). ^13^C NMR (201 MHz, DMSO-*d*_6_): δ = 165.7, 149.4, 145.8, 131.7, 129.4, 128.2, 120.8, 120.3, 117.1, 111.1, 108.7, 20.2. HRMS (ESI): m/z calculated for C_12_H_10_N_2_S (M+H)^+^: 215.0637; found: 215.0635.

**2-Methylnaphtho[1,2-*d*]thiazol-9-amine (SKA-178).** SKA-178 was prepared from 2-methyl-9-nitronaphtho[1,2-*d*]thiazole (SKA-216) in 2 steps according to General methods I and II as green crystals (39 mg, 17%); m.p = 87-88°C. ^1^H NMR (800 MHz, CDCl_3_): *δ* = 8.01 (d, *J* = 8.7 Hz, 1H), 7.76 (d, *J* = 8.7 Hz, 1H), 7.34 (t, J = 7.7 Hz, 1H), 7.22–7.19 (m, 1H), 7.11 (s, 2H), 6.86 (dd, J = 7.6, 1.1 Hz, 1H), 2.97 (s, 3H). ^13^C NMR (201 MHz, DMSO-*d*_6_): δ = 164.9, 148.6, 145.7, 134.1, 130.7, 127.4, 126.4, 119.5, 115.4, 115.1, 109.5, 20.2. HRMS (ESI): m/z calculated for C_12_H_10_N_2_S (M+H)^+^: 215.0637; found: 215.0635.

**4-(4-Methylnaphthalen-1-yl)thiazol-2-amine (SKA-190).** SKA-190 was prepared from 1-methylnaphthalene in 3 steps according to General methods III, IV, and V. The product was obtained as golden crystals (355 mg, 21%); m.p = 168–169°C (CAS 332064-25-2). ^1^H NMR (800 MHz, DMSO-*d*_6_): δ = 8.49 (dd, *J* = 8.4, 1.3 Hz, 1H), 8.09–8.06 (m, 1H), 7.60 (ddd, *J* = 8.3, 6.7, 1.4 Hz, 1H), 7.57–7.53 (m, 2H), 7.40 (dd, *J* = 7.2, 1.1 Hz, 1H), 7.12 (s, 2H), 6.74 (s, 1H), 2.70 (d, *J* = 1.0 Hz, 3H). ^13^C NMR (201 MHz, DMSO-*d*_6_): δ = 168.6, 150.3, 135.5, 132.9, 131.9, 131.8, 128.9, 128.4, 127.6, 126.8, 124.5, 124.3, 102.3, 21.7.

**4-(7-Methylnaphthalen-1-yl)thiazol-2-amine (SKA-193).** SKA-193 was prepared from 2-methylnaphthalene in 3 steps according to General methods III, IV, and V. The product was isolated as light yellow powder (70 mg, 4%); m.p. = 154–156°C. ^1^H NMR (800 MHz, CDCl_3_): δ = 8.06 (d, *J* = 1.8 Hz, 1H), 7.82 (d, *J* = 8.2 Hz, 1H), 7.79 (d, *J* = 8.3 Hz, 1H), 7.60 (dd, *J* = 7.0, 1.2 Hz, 1H), 7.43 (dd, *J* = 8.1, 7.0 Hz, 1H), 7.35 (dd, *J* = 8.3, 1.6 Hz, 1H), 6.64 (s, 1H), 5.41 (d, *J* = 150.2 Hz, 2H), 2.52 (s, 3H). ^13^C NMR (201 MHz, DMSO-*d*_6_): δ = 168.3, 150.6, 135.4, 133.3, 132.2, 131.4, 128.4, 128.3, 128.1, 127.2, 125.4, 124.9, 105.2, 22.2. HRMS (ESI): m/z calculated for C_14_H_12_N_2_S (M+H)^+^: 241.0794; found: 241.0793.

**4-(6-Methylnaphthalen-2-yl)thiazol-2-amine (SKA-198).** SKA-198 was prepared from 2-methylnaphthalene in 3 steps according to General methods III, IV, and V. The product was isolated as light yellow powder (110 mg, 7%); m.p. = 178–179°C. ^1^H NMR (800 MHz, DMSO-*d*_6_): δ = 8.31–8.28 (m, 1H), 7.94 (dd, *J* = 8.6, 1.7 Hz, 1H), 7.83 (dd, *J* = 24.8, 8.5 Hz, 2H), 7.69 (s, 1H), 7.38 (dd, *J* = 8.4, 1.7 Hz, 1H), 7.15 (d, *J* = 14.1 Hz, 3H), 2.55 (s, 3H). ^13^C NMR (201 MHz, DMSO-*d*_6_): δ = 168.6, 150.3, 135.5, 132.9, 131.9, 131.8, 128.9, 128.4, 127.6, 126.8, 124.5, 124.3, 102.3, 21.7. HRMS (ESI): m/z calculated for C_14_H_12_N_2_S (M+H)^+^: 241.0794; found: 241.0792.

***N*,5-Dimethylnaphtho[1,2-*d*]thiazol-2-amine (SKA-204).** SKA-204 was prepared from 2-Methyl-1-naphthylamine and methylisothiocyanate according to General Method VIII. The product was isolated as a white solid (35%). m.p = 172–173°C (CAS 1369251-74-0). ^1^H NMR (800 MHz, DMSO-*d*_6_): δ = 8.50–8.48 (m, 1H), 8.04–8.00 (m, 2H), 7.70 (d, *J* = 1.2 Hz, 1H), 7.58 (dddd, *J* = 20.1, 8.2, 6.7, 1.4 Hz, 2H), 3.05 (d, *J* = 4.7 Hz, 3H), 2.67 (s, 3H). ^13^C NMR (201 MHz, DMSO-*d*_6_): δ = 167.3, 146.7, 130.6, 126.5, 126.0, 125.3, 125.0, 124.4, 124.0, 123.98, 119.3, 30.7, 19.0.

**2-Methyl-6-nitronaphtho[1,2-*d*]thiazole (SKA-215).** SKA-215 was prepared from 1 g of 2-methyl-β-naphthothiazole according to General Method I. The product was isolated as light-yellow powder (200 mg, 6%); m.p. = 178–179°C. ^1^H NMR (800 MHz, DMSO-d_6_): δ = 9.15 (dt, *J* = 8.2, 1.1 Hz, 1H), 8.49 (dd, *J* = 9.2, 0.8 Hz, 1H), 8.29 (dd, *J* = 7.6, 1.3 Hz, 1H), 8.09 (d, J = 9.2 Hz, 1H), 7.74 (t, *J* = 7.9 Hz, 1H), 2.99 (s, 3H). ^13^C NMR (201 MHz, CDCl_3_): δ = 167.6, 149.3, 147.0, 133.1, 130.2, 129.3, 125.2, 123.5, 123.5, 122.4, 119.6, 20.2. HRMS (ESI): m/z calculated for C_12_H_8_N_2_O_2_S (M+H)^+^: 245.0379; found: 245.0376.

**2-Methyl-9-nitronaphtho[1,2-*d*]thiazole (SKA-216).** SKA-216 was prepared from 1 g of 2-methyl-β-naphthothiazole according to General Method I. The product was isolated as light-yellow crystals (280 mg, 22%); m.p. = 151–152°C. ^1^H NMR (800 MHz, DMSO-*d*_6_): δ = 8.10 (d, *J* = 8.0 Hz, 1H), 8.01 (d, *J* = 8.7 Hz, 1H), 7.85 (d, *J* = 8.7 Hz, 1H), 7.72 (d, *J* = 7.4 Hz, 1H), 7.60 (t, *J* = 7.8 Hz, 1H), 2.92 (s, 3H). ^13^C NMR (201 MHz, DMSO-*d*_6_): δ = 167.6, 146.9, 144.3, 135.4, 133.0, 131.9, 125.8, 125.6, 122.2, 121.6, 117.2, 20.4. HRMS (ESI): m/z calculated for C_12_H_8_N_2_O_2_S (M+H)^+^: 245.0379; found: 245.0376.

**5,6-Dinitronaphtho[1,2-*d*]thiazol-2-amine (SKA-218).** SKA-218 was prepared from 1 g of 2-methyl-β-naphthothiazole according to General Method I. The product was isolated as light brownish crystals (210 mg, 13.3%): m.p. = 172°C. ^1^H NMR (800 MHz, DMSO-*d*_6_): δ = 9.31 (s, 1H), 9.15 (dd, *J* = 8.2, 1.2 Hz, 1H), 8.50 (dd, *J* = 7.6, 1.2 Hz, 1H), 8.04 (dd, *J* = 8.2, 7.6 Hz, 1H), 3.02 (s, 3H). ^13^C NMR (201 MHz, DMSO-*d*_6_): δ = 175.7, 152.1, 145.9, 141.5, 131.8, 130.4, 128.8, 128.6, 126.9, 123.8, 114.7, 20.8. HRMS (ESI): m/z calculated for C_12_H_7_N_3_O_4_S (M+H)^+^: 290.02; found: 290.02.

**5-Bromo-4-(naphthalen-2-yl)thiazol-2-amine (SKA-220).** 2-Amino-4-(2-naphthy)thiazole (106 mg, 0.46 mmol) was dissolved in 10 ml of chloroform. Liquid bromine (30 μl, 0.46 mmol) in chloroform (3 ml) was then added drop-wise. The reaction mixture was stirred at room temperature for 30 min. The progress of the reaction was monitored by TLC. The reaction mixture was quenched with 4N NaOH and washed with sodium thiosulfate, water and brine. The organic phase was dried with anhydrous sodium sulfate and the solvent was evaporated under vacuum. The crude product was recrystallized from methanol. The product was isolated as light brown crystals (100 mg, 40%); m.p. = 140–141°C (CAS 99514-91-7). ^1^H NMR (800 MHz, DMSO-d_6_): δ = 8.35 (s, 1H), 7.96 (s, 2H), 7.99–7.91 (m, 2H), 7.56–7.52 (m, 2H), 7.38 (s, 2H). ^13^C NMR (201 MHz, DMSO-*d*_6_): δ = 167.4, 147.5, 133.0, 132.7, 131.7, 128.6, 128.0, 127.9, 127.5, 126.96, 126.9, 126.1, 88.0.

**4,4'-(1,4-Phenylene)bis(thiazol-2-amine) (SKA-230).** SKA-230 was prepared from 1,4-acetylbenzene in 2 steps according to general method IV, V. The product was isolated as light-yellow powder (197 mg, 47%); m.p. = 350°C (CAS 13355-22-1). ^1^H NMR (800 MHz, DMSO-d_6_): δ = 8.59 (s, 4H), 7.89 (s, 4H), 7.35 (s, 2H). ^13^C NMR (201 MHz, DMSO-d_6_): δ = 170.3, 128.0, 126.6, 119.9, 103.9.

**4-(Naphthalen-1-yl)-*N*-(3-(trifluoromethyl)phenyl)thiazol-2-amine (SKA-251).** SKA-251 was prepared from 1-acetonaphthone (500 mg, 2.94 mmol) and 3-(trifluoromethyl)phenylthiourea in 2 steps according to General methods IV and V. The product was isolated as white crystals (50 mg, 4.6%); m.p. = 135°C. ^1^H NMR (800 MHz, DMSO-d_6_): δ = 10.73 (s, 1H), 8.55 (d, *J* = 8.5 Hz, 1H), 8.38 (d, *J* = 2.1 Hz, 1H), 8.01 (m, 2H), 7.77 (ddd, *J* = 16.4, 7.6, 1.7 Hz, 2H), 7.56 (m, 4H), 7.27 (m, 2H). ^13^C NMR (201 MHz, DMSO-d_6_): δ = 162.7, 150.7, 142.2, 134.0, 133.1, 131.1, 130.5, 128.9, 128.7, 127.4, 126.6, 126.4, 126.2, 125.9, 120.7, 117.7, 117.6, 113.0, 113.0, 108.0. HRMS (ESI): m/z calculated for C_20_H_13_F_3_N_2_S(M+H)^+^: 371.0825; found: 371.0832.

**4-(Naphthalen-1-yl)-*N*-(pyridin-2-yl)thiazol-2-amine (SKA-258).** SKA-258 was prepared from 1-acetonaphthone and 2-pyridylthiourea in 2 steps according to General methods IV and V. The product was isolated as white powder (436 mg, 35%); m.p. = 225°C. ^1^H NMR (800 MHz, DMSO-d_6_): δ = 11.51 (s, 1H), 8.53 (m, 1H), 8.39 (ddd, *J* = 5.0, 1.9, 0.9 Hz, 1H), 7.99 (m, 1H), 7.96 (m, 1H), 7.75 (m, 2H), 7.59 (m, 3H), 7.26 (s, 1H), 7.15 (dt, *J* = 8.3, 1.0 Hz, 1H), 6.99 (ddd, *J* = 7.2, 5.0, 1.0 Hz, 1H).^13^C NMR (201 MHz, DMSO-d_6_): δ = 159.6, 152.3, 149.2, 147.0, 138.3, 134.0, 133.7, 131.1, 128.6, 128.5, 127.3, 126.5, 126.5, 126.3, 125.9, 116.4, 111.2, 110.1. HRMS (ESI): m/z calculated for C_18_H_13_N_3_S (M+H)^+^: 304.0903; found: 304.0905.

**4-(*p*-Tolyl)-*N*-(3-(trifluoromethyl)phenyl)thiazol-2-amine (SKA-260).** SKA-260 was prepared from 2-bromo-4’-methylacetophenone (500 mg, 2.34 mmol) and 3-(trifluoromethyl)phenylthiourea according to General Method V. The product was isolated as orange crystals (744 mg, 95%); m.p. = 108°C (CAS 778566-59-9). ^1^H NMR (800 MHz, DMSO-d_6_): δ = 10.68 (s, 1H), 8.42 (t, *J* = 2.0 Hz, 1H), 7.89 (dd, *J* = 8.2, 2.2 Hz, 1H), 7.85 (m, 2H), 7.61 (t, *J* = 7.9 Hz, 1H), 7.39 (s, 1H), 7.33 (m, 1H), 7.29 (m, 2H), 2.37 (s, 3H). ^13^C NMR (201 MHz, DMSO-d_6_): δ = 162.9, 150.5, 142.2, 137.5, 132.1, 130.6, 130.2, 130.1, 129.7, 125.9, 125.4, 124.1, 120.6, 117.6, 113.1, 103.3, 21.2.

**4-Phenyl-*N*-(pyridin-2-yl)thiazol-2-amine (SKA-265).** SKA-265 was prepared from 2-bromoacetophenone (500 mg, 2.5 mmol) and 2-pyridylthiourea according to General Method V. The product was isolated as white crystals (495 mg, 78%); m.p. = 158°C (CAS 92663-22-4). ^1^H NMR (800 MHz, DMSO-d_6_),: δ = 11.41 (s, 1H), 8.32 (ddd, *J* = 5.0, 1.9, 0.9 Hz, 1H), 7.92 (m, 2H), 7.72 (ddd, *J* = 8.4, 7.1, 1.9 Hz, 1H), 7.43 (m, 3H), 7.31 (td, *J* = 7.3, 1.3 Hz, 1H), 7.11 (dd, J = 8.3, 1.0 Hz, 1H), 6.94 (ddd, *J* = 7.2, 5.0, 1.0 Hz, 1H). ^13^C NMR (201 MHz, DMSO-d_6_): δ = 159.9, 152.3, 149.0, 146.9, 138.3, 135.2, 129.0, 127.9, 126.0, 116.4, 111.2, 106.3, 40.3, 39.7.

**4-Phenyl-*N*-(4-(trifluoromethyl)phenyl)thiazol-2-amine (SKA-268).** SKA-268 was prepared from 2-bromoacetophenone (500 mg, 2.5 mmol) and [4-(trifluoromethyl)phenyl]thiourea according to General Method V. The product was isolated as yellow crystals (530 mg, 66%); m.p. = 153°C (CAS 1303995-45-0). ^1^H NMR (800 MHz, DMSO-d_6_): δ = 10.75 (s, 1H), 8.01 (m, 4H), 7.75 (d, *J* = 8.5 Hz, 2H), 7.49(m, 3H), 7.37 (m, 1H). ^13^C NMR (201 MHz, DMSO-d_6_): δ = 162.8, 150.6, 144.8, 134.7, 129.1, 128.2, 126.8, 126.8, 126.2, 125.8, 124.4, 123.1, 116.9, 104.6.

**5-Ethylnaphtho[1,2-*d*]thiazol-2-amine (SKA-306).** SKA-306 was prepared from 1-ethylnaphthalene in 3 steps according to General methods I, II, and VII. The product was isolated as a brownish powder. (154 mg, 22%); m.p = 144–145°C. ^1^H NMR (800 MHz, DMSO-*d*_6_): δ = 8.39 (d, *J* = 7.7 Hz, 1H), 8.04 (d, *J* = 1.7 Hz, 1H), 7.66 (s, 1H), 7.54–7.48 (m, 4H), 3.11–3.01 (m, 2H), 1.31 (t, *J* = 7.5 Hz, 3H). ^13^C NMR (201 MHz, DMSO-*d*_6_): δ = 167.2, 146.9, 133.2, 130.2, 126.5, 125.7, 125.4, 125.2, 124.5, 124.4, 118.3, 25.7, 15.7. HRMS (ESI): m/z calculated for C_13_H_12_N_2_S (M+H)^+^: 229.0794; found: 229.0794.

**5-Propylnaphtho[1,2-*d*]thiazol-2-amine (SKA-307).** SKA-307 was prepared from 1-allylnaphthalene in 3 steps according to General Method I, II, and VII. The product was isolated as white crystals (495 mg, 70%); m.p. = 147–150°C. ^1^H NMR (800 MHz, DMSO-*d*_6_): δ = 8.41–8.33 (m, 1H), 8.07–7.98 (m, 1H), 7.65 (s, 1H), 7.56–7.45 (m, 4H), 3.03–2.96 (m, 2H), 1.70 (q, *J* = 7.5 Hz, 2H), 0.98 (t, *J* = 7.3 Hz, 3H).^13^C NMR (201 MHz, DMSO-*d*_6_): δ = 167.2, 147.2, 131.5, 130.3, 126.6, 125.6, 125.3, 125.2, 124.6, 124.5, 119.3, 34.9, 24.2, 14.5. HRMS (ESI): m/z calculated for C_14_H_14_N_2_S (M+H)^+^: 243.0950; found: 243.0950.

**5-Isopropylnaphtho[1,2-*d*]thiazol-2-amine (SKA-308).** SKA-308 was prepared from 1-isopropylnaphthalene in 3 steps according to general method I, II, and VII. The product was isolated as white powder (179 mg, 10%); m.p. = 179–181°C.^1^H NMR (800 MHz, DMSO-*d*_6_): δ = 8.47–8.41 (m, 1H), 8.18–8.13 (m, 1H), 7.77 (s, 1H), 7.59–7.52 (m, 4H), 1.38 (d, *J* = 6.8 Hz, 7H). ^13^C NMR (201 MHz, DMSO-*d*_6_): δ = 167.3, 146.9, 137.6, 130.3, 129.8, 126.6, 125.5, 125.4, 124.7, 124.0, 115.5, 28.4, 24.0. HRMS (ESI): m/z calculated for C_14_H_14_N_2_S (M+H)^+^: 243.0950; found: 243.0949.

**Naphtho[1,2-*d*:5,6-*d’*]bis(thiazole)-2,7-diamine (SKA-318).** 1,5-Diaminonaphthalene (500 mg, 3.16 mmol) and ammonium thioscyanate (480 mg, 6.32 mmol) were dissolved in 20 mL of anhydrous acetonitrile at room temperature. Benzyl trimethyl ammonium tribromide (2.48 g, 6.36 mmol) was added to the solution, and the reaction mixture was stirred at room temperature for 5 h. After completion of the reaction as determined by TLC, the reaction mixture was concentrated to dryness under vacuum, and the residue was diluted with 100 ml of water. The pH of the solution was adjusted to between 7 to 7.5 with saturated sodium bicarbonate solution. The product was isolated as a red crystal (20 mg, 2.5%); m.p = 194°C (CAS 1621401-78-2). ^1^H NMR (800 MHz, DMSO-*d*_6_): δ = 7.41 (d, *J* = 9.0 Hz, 2H), 7.38 (d, *J* = 9.0 Hz, 2H), 5.80 (s, 4H). ^13^C NMR (201 MHz, DMSO-*d*_6_): δ = 168.1,141.8, 129.2, 123.4, 112.3, 102.7.

**5-Nitronaphtho[1,2-*d*]thiazol-2-amine (SKA-321).** SKA-321 was prepared from 1-naphtho[1,2-*d*]thiazol-2-amine (500 mg, 7 mmol) according to General Method I. The product was isolated as white powder (30 mg, 2%); m.p. = 289–291°C (CAS 320340-92-9) ^1^H NMR (800 MHz, DMSO-*d*_6_): δ = 8.95 (s, 1H), 8.67 (dt, *J* = 8.7, 0.9 Hz, 1H), 8.54 (dd, *J* = 8.3, 1.6 Hz, 1H), 8.40 (s, 2H), 7.76 (ddd, *J* = 8.6, 6.8, 1.4 Hz, 1H), 7.69 (ddd, *J* = 8.1, 6.8, 1.1 Hz, 1H). ^13^C NMR (201 MHz, DMSO-*d*_6_): δ = 173.2, 155.2, 138.5, 129.3, 127.1, 125.2, 125.1, 124.8, 123.7, 121.4.

**5-Phenylnaphtho[1,2-*d*]thiazol-2-amine (SKA-326).** SKA-326 was prepared from 1-phenylnaphthalene in 3 steps according to General Method I, II, and VII. The product was isolated as white crystals (495 mg, 78%); m.p. = 177–178°C ^1^H NMR (800 MHz, DMSO-*d*_6_): δ = 8.43 (d, 1H), 7.78 (dt, *J* = 8.5, 0.9 Hz, 1H), 7.74 (s, 1H), 7.68 (s, 2H), 7.57–7.50 (m, 3H), 7.50–7.46 (m, 2H), 7.48–7.42 (m, 2H).^13^C NMR (201 MHz, DMSO-*d*_6_): δ = 168.1, 148.2, 140.8, 133.0, 130.4, 130.0, 128.8, 127.5, 126.3, 126.1, 126.0, 125.7, 125.3, 124.4, 120.5. HRMS (ESI): m/z calculated for C_17_H_12_N_2_S (M+H)^+^: 277.0794; found: 277.0798.

***N^5^,N^5^*-Diethylnaphtho[1,2-*d*]thiazole-2,5-diamine (SKA-330).** SKA-330 was prepared from diethylamine hydrochloride in 3 steps according to General Method II, VI, and VII. The product was isolated as yellowish crystals (50 mg, 8%); m.p. = 119–121°C.^1^H NMR (800 MHz, DMSO-*d*_6_): δ = 8.32 (d, 1H), 8.27 (d, 1H), 7.60 (s, 1H), 7.50 (ddd, *J* = 8.1, 6.7, 1.3 Hz, 1H), 7.45 (ddd, *J* = 8.2, 6.7, 1.4 Hz, 1H), 7.43 (s, 2H), 3.09 (q, *J* = 7.1 Hz, 4H), 0.95 (t, *J* = 7.1 Hz, 6H). ^13^C NMR (201 MHz, DMSO-*d*_6_): δ = 167.0, 145.0, 141.6, 130.3, 126.9, 125.9, 125.4, 124.9, 124.59, 124.1, 112.7, 48.4, 21.5, 12.9. HRMS (ESI): m/z calculated for C_15_H_17_N_3_S (M+H)^+^: 272.1216; found: 272.1217.

***N^5^,N^5^*-Dimethylnaphtho[1,2-*d*]thiazole-2,5-diamine (SKA-331).** SKA-331 was prepared from dimethylamine hydrochloride in 3 steps according to General Method II, VI, and VII. The product was isolated as white crystals (30 mg, 5%); m.p. = 171–174°C. ^1^H NMR (800 MHz, DMSO-*d*_6_): δ = 8.32 (d, 1H), 8.17 (d, 1H), 7.51 (d,1H), 7.51 (s, 1H), 7.48 (d,1H), 7.42 (s, 2H), 2.78 (s, 6H). ^13^C NMR (201 MHz, DMSO-*d*_6_): δ = 166.7, 145.2, 144.4, 127.5, 127.0, 126.0, 125.4, 124.9, 124.5, 124.3, 108.8, 45.7 HRMS (ESI): m/z calculated for C_13_H_13_N_3_S (M+H)^+^: 244.0903; found: 244.0902.

**5-(1*H*-Imidazol-1-yl)naphtho[1,2-*d*]thiazol-2-amine (SKA-334).** SKA-334 was prepared from imidazole in 3 steps according to General Method II, VI, and VII. The product was isolated as brownish powder (130 mg, 19%); m.p. = 272–275°C. ^1^H NMR (800 MHz, DMSO-*d*_6_): δ = 8.46 (d, 1H), 7.99 (s, 1H), 7.94 (t, J = 1.1 Hz, 1H), 7.85 (s, 2H), 7.62 (ddd, J = 8.1, 6.8, 1.1 Hz, 1H), 7.55–7.50 (m, 1H), 7.33 (dt, J = 8.4, 0.9 Hz, 2H), 7.18 (t, J = 1.1 Hz,1H). ^13^C NMR (201 MHz, DMSO-*d*_6_): δ = 169.3, 149.1, 139.3, 129.1, 128.4, 127.2, 126.9, 126.8, 125.8, 124.5, 124.4, 123.0, 122.6, 118.6. HRMS (ESI): m/z calculated for C_14_H_10_N_4_S (M+H)^+^: 267.0699; found: 267.0700.

**5-(1*H*-Pyrazol-1-yl)naphtho[1,2-*d*]thiazol-2-amine (SKA-335).** SKA-335 was prepared from pyrazole in 3 steps according to General methods II, VI, and VII. The product was isolated as brown crystal (220 mg, 31%); m.p. = 226–227°C. ^1^H NMR (800 MHz, DMSO-*d*_6_): δ = 8.44 (dt, *J* = 8.3, 1.1 Hz, 1H), 8.12 (d, *J* = 2.3 Hz, 1H), 7.97 (s, 1H), 7.85–7.80 (m, 3H), 7.60 (ddd, *J* = 8.2, 4.7, 3.2 Hz, 1H), 7.52–7.47 (m, 2H), 6.57 (t, *J* = 2.1 Hz, 1H). ^13^C NMR (201 MHz, DMSO-*d*_6_): δ = 169.2, 149.0, 140.6, 133.2, 130.8, 128.2, 126.7, 126.5, 125.9, 124.3, 124.2, 123.6, 118.1, 106.7. HRMS (ESI): m/z calculated for C_14_H_10_N_4_S (M+H)^+^: 267.0699; found: 267.0700.

**5-(1*H*-1,2,3-Triazol-1-yl)naphtho[1,2-*d*]thiazol-2-amine (SKA-339).** SKA-339 was prepared from 1,2,3-triazole in 3 steps according to General methods II, VI, and VII. The product was isolated as purplish crystals (120 mg, 17%); m.p = 208°C. ^1^H NMR (800 MHz, DMSO-*d*_6_): δ = 8.48 (d, Hz,1H), 8.20 (s, 2H), 8.15 (s, 1H), 7.91 (s, 2H), 7.66–7.61 (m, 2H), 7.54 (ddd, *J* = 8.3, 6.7, 1.3 Hz, 1H). ^13^C NMR (201 MHz, DMSO-*d*_6_): δ = 169.8, 149.7, 136.3, 130.2, 126.9, 126.9, 126.8, 125.7, 124.4, 124.2, 123.3, 118.2. HRMS (ESI): m/z calculated for C_13_H_9_N_5_S (M+H)^+^: 268.0651; found: 268.0654.

**5-(3-Methyl-1*H*-pyrazol-1-yl)naphtho[1,2-*d*]thiazol-2-amine (SKA-340).** SKA-340 was prepared from 3-methylpyrazole in 3 steps according to General methods II, VI, and VII. The product was isolated as brownish crystals (140 mg, 19%); m.p = 189–190°C. ^1^H NMR (800 MHz, DMSO-*d*_6_): δ = 8.46–8.41 (m, 1H), 7.97 (d, *J* = 2.2 Hz, 1H), 7.92 (s, 1H), 7.81 (s, 2H), 7.61–7.57 (m, 2H), 7.50 (ddd, *J* = 8.4, 7.0, 1.3 Hz, 1H), 6.35 (d, *J* = 2.2 Hz, 1H), 2.31 (s, 3H). ^13^C NMR (201 MHz, DMSO-*d*_6_): δ = 169.1, 149.0, 148.7, 133.8, 131.0, 128.1, 126.6, 126.4, 125.9, 124.3, 124.2, 123.8, 117.9, 106.4,13.9. HRMS (ESI): m/z calculated for C_15_H_12_N_4_S (M+H)^+^: 281.0855; found: 281.0858.

**5-(3-(Trifluoromethyl)-1*H*-pyrazol-1-yl)naphtho[1,2-*d*]thiazol-2-amine (SKA-341).** SKA-341 was prepared from 3-(trifluoromethyl)pyrazole in 3 steps according to General methods II, VI, and VII. The product was isolated as white crystals (150 mg, 17%); m.p = 206–207°C. ^1^H NMR (800 MHz, DMSO-*d*_6_): δ = 8.47 (dt, *J* = 8.3, 1.0 Hz, 1H), 8.41 (dd, *J* = 2.4, 1.1 Hz, 1H), 8.11 (s, 1H), 7.91 (s, 2H), 7.63 (ddd, *J* = 8.2, 6.7, 1.2 Hz, 1H), 7.55 (ddd, *J* = 8.3, 6.8, 1.3 Hz, 1H), 7.37 (dt, *J* = 8.5, 0.9 Hz, 1H), 7.07 (d, *J* = 2.4 Hz, 1H).^13^C NMR (201 MHz, DMSO-*d*_6_): δ = 169.8, 149.8, 142.4, 136.0, 129.4, 127.8, 127.1, 126.9, 125.7, 124.5, 124.1, 122.7, 122.6, 118.9, 105.5. HRMS (ESI): m/z calculated for C_15_H_9_F_3_N_4_S (M+H)^+^: 335.0573; found: 335.0575.

**5-(4-Methyl-1*H*-imidazol-1-yl)naphtho[1,2-*d*]thiazol-2-amine (SKA-343).** SKA-343 was prepared from 4-methylimidazole in 3 steps according to General methods II, VI, and VII. The product was isolated as brownish crystals (160 mg, 22%); m.p = 269–271°C. ^1^H NMR (800 MHz, DMSO-*d*_6_): δ = 8.45 (d, 1H), 7.95 (d,1H), 7.86 (s, 1H), 7.82 (s, 2H), 7.77 (d, 1H), 7.63–7.58 (m, 1H), 7.52 (dtd, *J* = 7.6, 6.5, 1.3 Hz, 1H), 7.39 (dt, *J* = 8.4, 0.9 Hz, 1H), 2.23 (d, *J* = 1.1 Hz, 3H). ^13^C NMR (201 MHz, DMSO-*d*_6_): δ = 169.4, 169.1, 148.9, 139.0, 138.5, 137.5, 128.4, 127.5, 126.8, 126.8, 126.8, 126.4, 125.8, 124.5, 124.4, 124.4, 122.8, 122.6, 119.9, 119.2, 118.3, 55.3, 14.1. HRMS (ESI): m/z calculated for C_15_H_12_N_4_S (M+H)^+^: 281.0855; found: 281.0859.

**5-(2-Methyl-1*H*-imidazol-1-yl)naphtho[1,2-*d*]thiazol-2-amine (SKA-344).** SKA-344 was prepared from 2-methylimidazole in 3 steps according to General methods II, VI, and VII. The product was isolated as light brown powder (300 mg, 41%); m.p = 264–265°C. ^1^H NMR (800 MHz, DMSO-*d*_6_): δ = 8.46 (ddd, *J* = 8.2, 1.2, 0.7 Hz, 1H), 7.97 (s, 1H), 7.87 (s, 2H), 7.61 (ddd, *J* = 8.1, 6.8, 1.2 Hz, 1H), 7.51 (ddd, *J* = 8.2, 6.8, 1.3 Hz, 1H), 7.28 (d, *J* = 1.3 Hz, 1H), 7.05 (dt, *J* = 8.4, 0.9 Hz, 1H), 7.02 (d, *J* = 1.3 Hz, 1H), 2.03 (s, 3H). ^13^C NMR (201 MHz, DMSO-*d*_6_): δ = 169.4, 149.3, 145.8, 128.8, 127.5, 127.2, 127.0, 126.8, 126.0, 124.5, 124.5, 123.1, 122.6, 119.5, 13.2. HRMS (ESI): m/z calculated for C_15_H_12_N_4_S (M+H)^+^: 281.0855; found: 281.0859.

**5-(4-Methyl-1*H*-pyrazol-1-yl)naphtho[1,2-*d*]thiazol-2-amine (SKA-345).** SKA-345 was prepared from 4-methylpyrazole in 3 steps according to General methods II, VI, and VII. The product was isolated as brownish powder (280 mg, 39%); m.p = 249°C. ^1^H NMR (800 MHz, DMSO-*d*_6_): δ = 8.43 (dt, *J* = 8.3, 0.9 Hz, 1H), 7.97 (s, 1H), 7.92 (s, 1H), 7.87 (t, *J* = 0.9 Hz, 1H), 7.81 (s, 2H), 7.62–7.54 (m, 2H), 7.49 (ddd, *J* = 8.3, 6.7, 1.3 Hz, 1H), 2.16 (s, 3H). ^13^C NMR (201 MHz, DMSO-*d*_6_): δ = 169.1, 148.8, 141.2, 131.6, 131.1, 128.1, 126.6, 126.4, 125.9, 124.3, 123.7, 117.8, 116.3, 9.2. HRMS (ESI): m/z calculated for C_15_H_12_N_4_S (M+H)^+^: 281.0855; found: 281.0857.

**5-(1*H*-1,2,4-Triazol-1-yl)naphtho[1,2-*d*]thiazol-2-amine (SKA-347).** SKA-347 was prepared from 1,2,4-triazole in 3 steps according to General methods II, VI, and VII. The product was isolated as light yellowish crystals (200 mg, 28%); m.p = 273°C. ^1^H NMR (800 MHz, DMSO-*d*_6_): δ = 8.97 (s, 1H), 8.47 (dt, *J* = 8.3, 1.0 Hz, 1H), 8.33 (s, 1H), 8.08 (s, 1H), 7.92 (s, 2H), 7.63 (ddd, *J* = 8.1, 6.8, 1.1 Hz, 1H), 7.54 (ddd, *J* = 8.3, 6.8, 1.3 Hz, 1H), 7.42 (dt, *J* = 8.5, 0.9 Hz, 1H). ^13^C NMR (201 MHz, DMSO-*d*_6_): δ = 169.7, 152.6, 149.8, 146.6, 127.8, 127.0, 126.9, 126.7, 125.84, 124.4, 124.1, 122.9, 118.8. HRMS (ESI): m/z calculated for C_13_H_9_N_5_S (M+H)^+^: 268.0651; found: 268.0655.

### Crystal Structure Determination

The SKA-218, SKA-339, SKA-340, SKA-343 and SKA347 crystals selected for data collection were mounted and optically centered in a nitrogen low temperature stream –183°C (90K), on the Bruker diffractometer with an APEX2 CCD detector or a Bruker D8 Venture diffractometer equipped with a Photon100 CMOS detector (Bruker, Madison, WI). Data were collected with the use of Mo Ka radiation in all cases (λ = 0.71073 Å). The structures were solved by direct methods (SHELXT) and refined by full-matrix least-squares on F^2^ (SHELXL-2018/3). All non-hydrogen atoms were refined with anisotropic displacement parameters. For a description of the method, see (Sheldrick, 2008).

**Crystal Data SKA-218, JF2786, APEX2,** C_12_H_7_N_3_O_4_S, F.W = 289.27, purple block, dimensions = 0.472 x 0.400 x 0.232 mm^3^, monoclinic, P2_1_/n, a = 9.9178(15) Å, b = 15.885(2) Å, c = 7.4528(11) Å, β = 93.4141(19)°, V = 1172.0(3) Å^3^, Z = 4, 182 parameters, 0 restraints.

**Crystal Data SKA-339, JF2770, D8 Venture,** C_13_H_9_N_5_S, F.W = 267.31, yellow plate, dimensions = 0.136 x 0.071 x 0.023 mm^3^, monoclinic, P2_1_/n, a = 16.3915(19) Å, b = 3.7972(4) Å, c = 19.437(2) Å, β = 112.065(2)°, V = 1121.2(2) Å^3^, Z = 4, 208 parameters, 0 restraints.

**Crystal Data SKA-340, JF2772, D8 Venture,** C_15_H_12_N_4_S, F.W = 280.35, yellow rod, dimension = 0.401 x 0.143 x 0.073 mm^3^, triclinic, P-1, a = 8.7703(10) Å, α = 96.219(4)°, b = 9.3235(11) Å, β = 103.017(4)°, c = 10.7112(13) Å, γ = 110.077(4)°, V = 784.83(16) Å^3^, Z = 2, 237 parameters, 0 restraints.

**Crystal Data SKA-343, JF2787, D8 Venture,** C_15_H_12_N_4_S F.W = 280.35, colorless rod, dimensions = 0.221 x 0.086 x 0.051 mm^3^, monoclinic, P2_1_/n, a = 15.9106(10) Å, b = 20.4896(13) Å, c = 7.9634(5) Å, β = 94.589(2)°, V = 2587.8(3) Å^3^, Z = 8, 458 parameters, 0 restraints.

**Crystal Data SKA-347, JF2769, D8 Venture,** C_13_H_9_N_5_S, F.W. = 267.31, colorless block, dimensions = 205 x 0.150 x 0.088 mm^3^, monoclinic P2_1_/n a = 3.7801(3) Å, b = 14.7453(12) Å, c = 20.2851(17) Å, β = 94.550(2)°, V = 1127.10(16) Å^3^, Z = 4, R1 = 0.0305, wR2 = 0.0797, 209 parameters, 0 restraints.

## Results

### Structure-Based Drug Design Using the Crystal Structure of the K_Ca_2.2 CaM-BD/CaM Interface

We previously generated homology models of K_Ca_3.1 and K_Ca_2.3 (Brown et al., 2017) using the Rosetta membrane method (Rohl et al., 2004; Bender et al., 2016; Alford et al., 2017) and the x-ray crystal structure of the K_Ca_2.2 CaM-BD/CaM (Zhang et al., 2013) as a template. We localized the binding site of the benzothiazoles/oxazoles to the CaM-BD/CaM interface and generate models of the K_Ca_3.1 and K_Ca_2.3 CaM-BD/CaM complexes with SKA-121 and SKA-111 using Rosetta Ligand docking (Meiler and Baker, 2006; Davis and Baker, 2009; Bender et al., 2016). The docking models of K_Ca_3.1 showed that the amino groups of the benzoxazole ring of SKA-121 and of the benzothiazole ring of SKA-111 form hydrogen bonds with M51 and E54 in calmodulin (Brown et al., 2017). Moreover, E54 was further stabilized by an extensive hydrogen bond network with R362, E295 and N300 in the K_Ca_3.1 channel, which we hypothesized to be responsible for the K_Ca_3.1 selectivity of SKA-121 and SKA-111. In the K_Ca_2.3 or K_Ca_2.2 model, however, SKA-111 and SKA-121 formed only hydrogen bonds with M51 and E54, due to the shorter length of the sidechain of S622 in K_Ca_2.3 or N474 in K_Ca_2.2 than that of the corresponding R362 in K_Ca_3.1 (**Supplementary Figure 1**).

**Figure 1:**
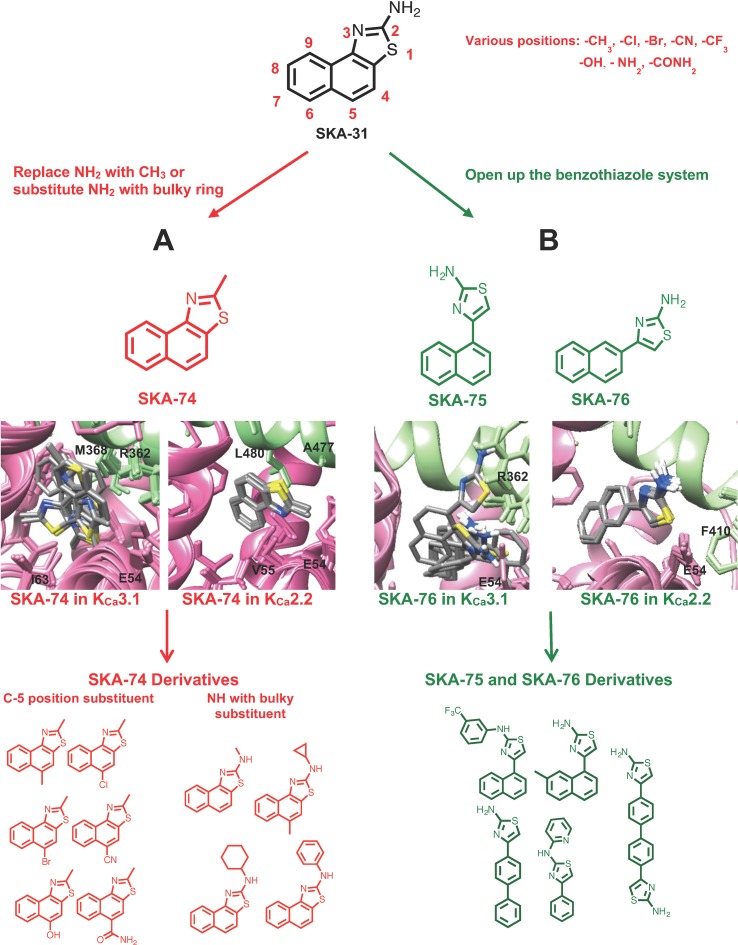
Design scheme of K_Ca_2.2 selective activators and Rosetta models of the top 10 binding poses with the lowest energy of template compounds in the interface between CaM (pink) and the CaM-BD (light green) of K_Ca_3.1 and K_Ca_2.2. The docking model of SKA-74 **(A)** and SKA-76 **(B)** showed that the ten lowest binding energy scored models exhibit good structural convergence in K_Ca_2.2 but not in K_Ca_3.1 suggesting selectivity for K_Ca_2.2 over K_Ca_3.1.

Using the information from these computational docking models, we here intended to design new K_Ca_2 selective activators by attempting to predict whether the compounds would show selectivity for K_Ca_2 over K_Ca_3.1 channels. However, instead of our previously generated K_Ca_2.3 CaM-BD/CaM homology model, we here used a K_Ca_2.2 CaM-BD/CaM model based on the K_Ca_2.2 CaM-BD/CaM-NS309 crystal structure (pdb:4J9Z). We made this switch because K_Ca_2.2 is the most abundantly expressed K_Ca_2 channel in the mammalian CNS (Adelman et al., 2012) and therefore constitutes an attractive target for the treatment of ataxia and epilepsy. We additionally docked eight more 2-amino-naphthobenzothiazole derivatives (SKA-31, SKA-44, SKA-45, SKA-72, SKA-73, SKA-107, SKA-117, and SKA-120) into the K_Ca_2.2 and K_Ca_3.1 homology models and found that these compounds exhibited the same hydrogen bond network as SKA-121 and SKA-111 in K_Ca_3.1 (**Supplementary Figure 1**). Based on these docking poses we hypothesized that disruption of the hydrogen bond between the –NH_2_ group of the benzothiazole ring, and the CaM M51 and E54 residues, which are present in both the K_Ca_3.1 and K_Ca_2.2 models, might be a way to achieve K_Ca_2.2 selectivity. Our goal here was to first “break” this hydrogen bond to ideally achieve K_Ca_2.2 selectivity and then regain potency by adding substituents in other positions to pick up unique contacts in K_Ca_2.2. We therefore virtually added various substituents in the C-4,5,6,7,8,9 positions of SKA-74 (**Figure 1A**), a compound which contains a methyl group in C-2 position instead of an -NH_2_ group and which we had previously found to activate K_Ca_2 and K_Ca_3.1 channels with a similar ∼30 μM potency (Coleman et al., 2014). To improve van der Waals contacts we introduced -CH_3_, -Br, -Cl, -CF_3_ groups. The C-5 position was chosen for the first trial since the K_Ca_2.2 docking model of SKA-74 showed that it is adjacent to A484 and in range for new interactions. We also virtually introduced larger substituents such as cyclohexyl, cyclopentyl, cyclopropyl and phenyl on the 2-position amino group because the K_Ca_2.2 model showed more space in this region of the binding site than the K_Ca_3.1 model. In addition, we also virtually generated double substituted SKA-74 derivatives (a bulky substituent in the C-2 position and methyl in C-5 position). In parallel we designed a small focused library of 2-aminothiazoles (SKA-75 and SKA-76 derivatives; **Figure 1B**). Our reasoning for the choice of these two compounds as additional templates was that SKA-75, like SKA-74, had previously been found to be of similar potency (∼30 μM) on both K_Ca_2.3 and K_Ca_3.1, and that SKA-76 was slightly more potent on K_Ca_2.3 (∼25 μM) than K_Ca_3.1 (∼50 μM) (Coleman et al., 2014). In addition, the molecular docking models of SKA-75 and SKA-76, which both lack the continuous conjugation between the naphthalene and the 2-aminothiazole ring (**Figure 1** right), showed that the ten lowest energy scored models exhibited good structural convergence in K_Ca_2.2 but not in K_Ca_3.1 (**Figure 1B**). In order to improve the potency and selectivity of SKA-75 and SKA-76 we virtually added a methyl group in the 2,3,4,5,6,7 or 8 positions of the naphthalene ring in SKA-75 and SKA-76. We then replaced the naphthalene ring with a bi-phenyl ring, a 2-aminophenylthiazole or a 2-aminobiphenylthiazole. We further replaced the naphthalene ring with a phenyl ring and added larger groups such as *N*-trifluorophenyl, *N*-pyridine to the -NH_2_ group of the 2-aminothiazole ring.

**Figure 2:**
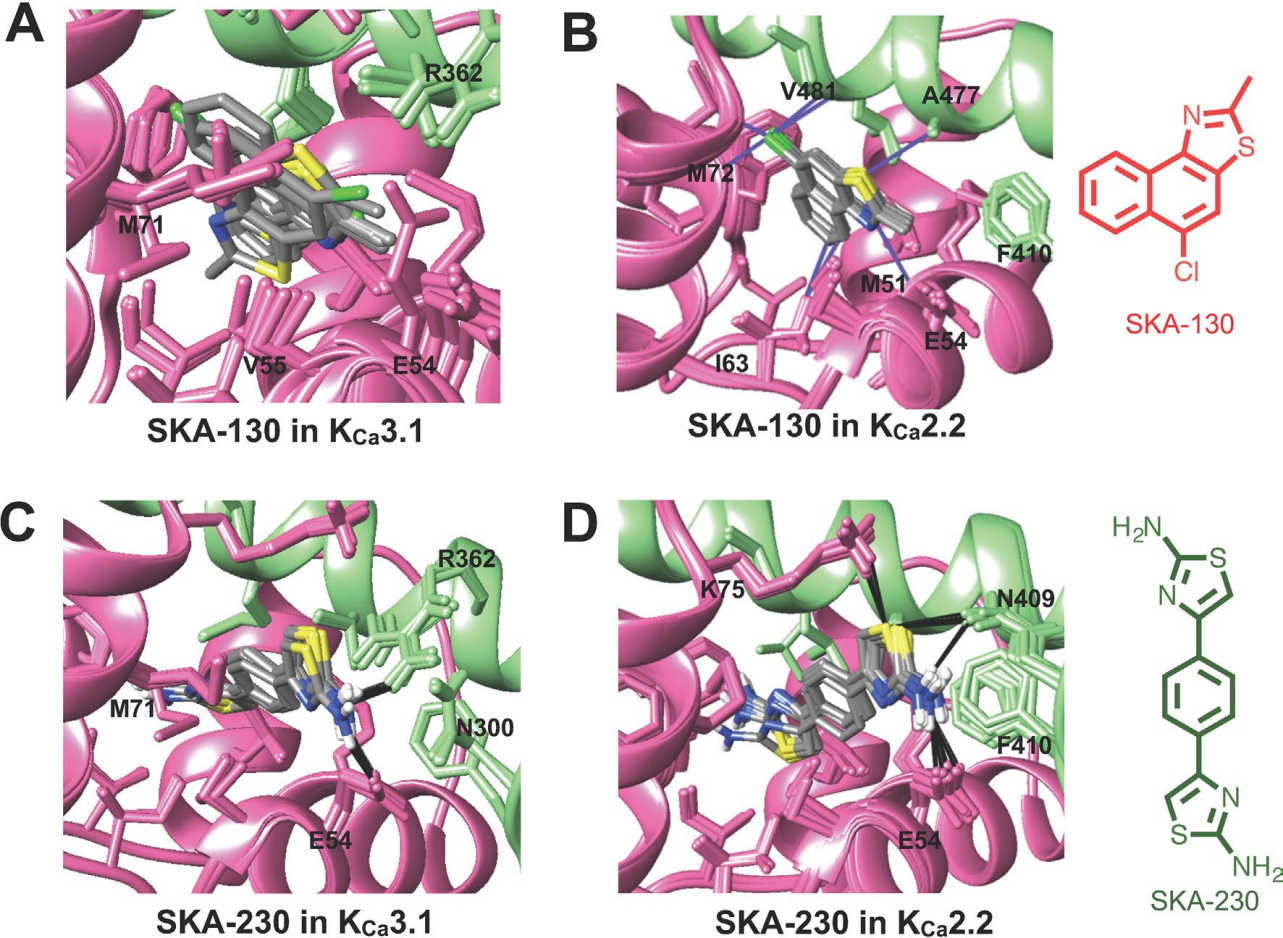
Rosetta ligand docking models of the lowest energy-binding poses of SKA-130 **(A**, **B)** and SKA-230 **(C**, **D)** in the interface between CaM (pink) and CaM-BD (light green) of K_Ca_3.1 and K_Ca_2.2. Hydrogen bonds and van der Waals interactions are indicated by purple and black lines. **(A**, **B)** Molecular docking suggests that SKA-130 converged well and formed several van der Waals contacts in K_Ca_2.2 but not in K_Ca_3.1. **(C**, **D)** Both K_Ca_3.1 and K_Ca_2.2 models exhibited good structural convergence. The molecular docking model showed that while SKA-230 only formed 2 hydrogen bonds in K_Ca_3.1, it formed four hydrogen bonds in K_Ca_2.2 suggesting selectivity for K_Ca_2.2.

The virtually proposed 63 SKA-74 derivatives, 87 SKA-75 and SKA-76 derivatives as well as another 18 SKA-31-related compounds (see **Supplementary Figure 2** for all structures) were randomly placed into the K_Ca_2.2 and K_Ca_3.1 homology models of the CaM-BD/CaM interface pocket, energy minimized through the three stages of the RosettaLigand method, and the top 10 lowest binding energy scoring models were analyzed. SKA-74 derivatives with –CH_3_, -CF_3_, -Br and –Cl in 5-position were predicted to show selectivity for K_Ca_2.2 channels over K_Ca_3.1 (**Figures 2A, B**) because they converged well in the K_Ca_2.2 model and made van der Waals interactions (dark purple in **Figure 2B**) with A477, V481 in K_Ca_2.2 and M72, F68, I63 and M51 in CaM. In contrast, the top 10 lowest energy models of all SKA-74 derivatives with bulky substituents in C-2 position did not converge in either K_Ca_2.2 or K_Ca_3.1, whereas all double substituted SKA 74 derivatives were predicted to be K_Ca_2.2 selective with good structural convergence in K_Ca_2.2. For the SKA-75 and SKA 76 derivatives, the docking model suggested that addition of a methyl group to the 6-position of the naphthalene ring in SKA 76 (SKA-198) created new hydrogen bonds and good structural convergence in the K_Ca_2.2 model but not in K_Ca_3.1. Replacement of the naphthalene ring of SKA-75 with a bi-phenyl ring, a 2-aminophenylthiazole or a 2-aminobiphenylthiazole (SKA-232, SKA-230 and SKA-255) created new hydrogen bonds and good structural convergence in both K_Ca_2.2 and K_Ca_3.1. The molecular docking model showed that while these SKA-75 derivatives (SKA-232, SKA-230 and SKA-255) only formed 2 hydrogen bonds in K_Ca_3.1, they formed four hydrogen bonds in K_Ca_2.2 (see **Figures 2C, D** for SKA-230) suggesting selectivity for K_Ca_2.2 as well as relatively high potency.

### Synthesis and Activity Testing of the Newly Designed K_Ca_ Channel Activators

**Figure 3:**
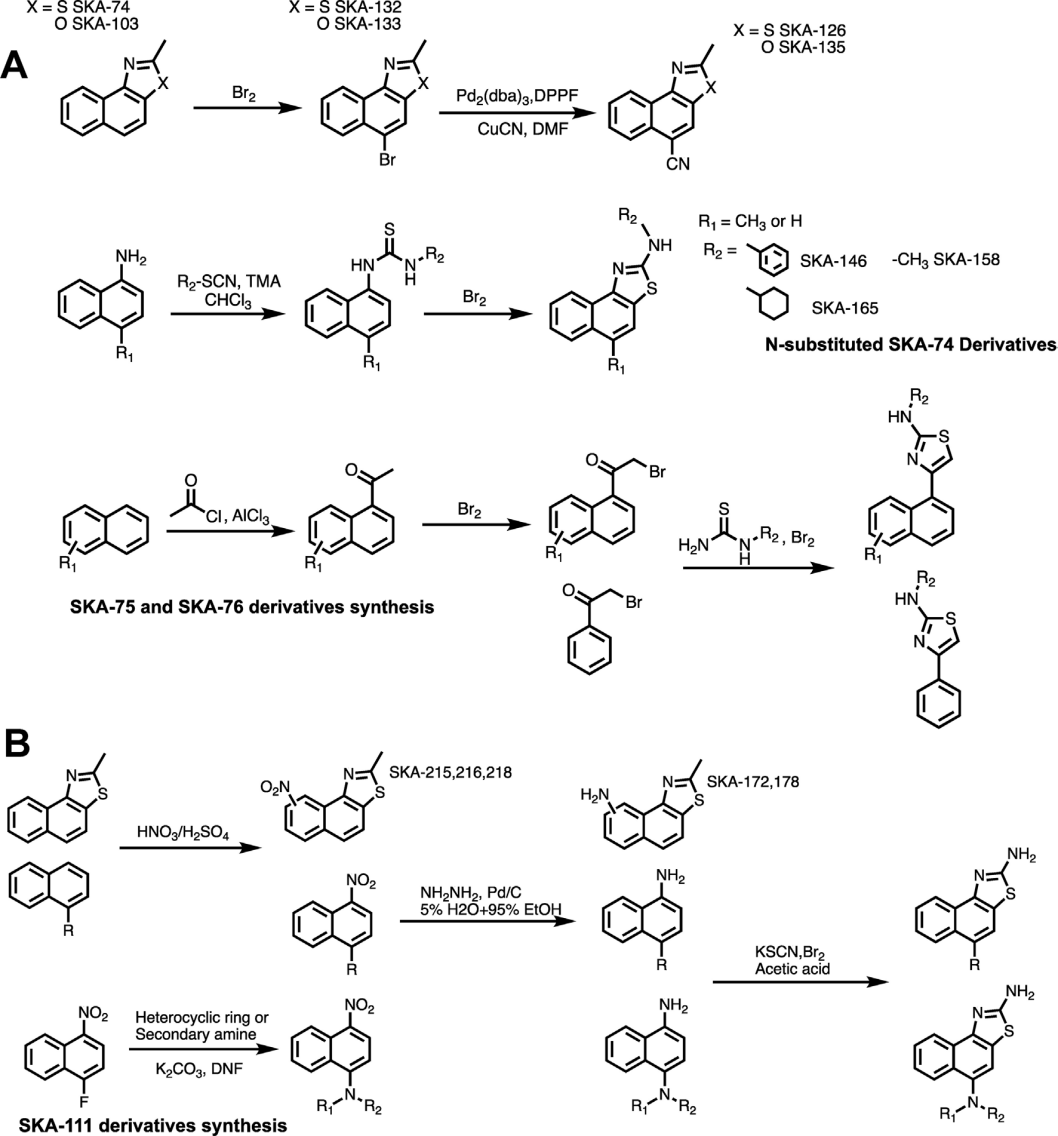
General scheme for the synthesis of SKA-74, SKA-75, SKA-76 **(A)** and SKA-111 derivatives **(B)**.

Based on the docking models we chose 26 (16 SKA-74 and 10 SKA-75/76 derivatives) of the 168 virtual compounds for synthesis including some that were not predicted to be selective in order to verify that the predicted selectivity in the model is consistent with experiments (see **Supplementary Figure 2** where the chosen structures are highlighted in color). A general scheme of the compound synthesis is given in **Figure 3**. To obtain 5-CH_3_ substituted SKA-74 derivatives the commercially available starting materials, 2-methylnaphtho[1,2-*d*]thiazole (SKA-74) and 2-methylnaphtho[1,2-*d*]oxazole (SKA-103), were first brominated using liquid bromine to obtain SKA-132 and SKA-133, which were then reacted with CuCN in a cross coupling reaction with a palladium catalyst to obtain SKA-126 and SKA-135. SKA-130 was synthesized by electrophilic aromatic substitution with *N*-chlorosuccinimide. *N*-substituted SKA-74 derivatives were prepared from 1-aminonaphthalene, which was reacted with isothiocyanates to the *N,N*’-disubstituted thioureas, which were then cyclized using bromine. SKA 75 and SKA-76 derivatives were synthesized starting from methyl naphthalene, 1,4 acetylbenzene, 1-acetonaphthone or 2-bromo substituted acetophenone. A classic Fridel-Craft acylation was used to synthesize monoacylated intermediates, which were then brominated on the alpha carbon followed by cyclization of the compounds with substituted thiourea and liquid bromine.

**Figure 4:**
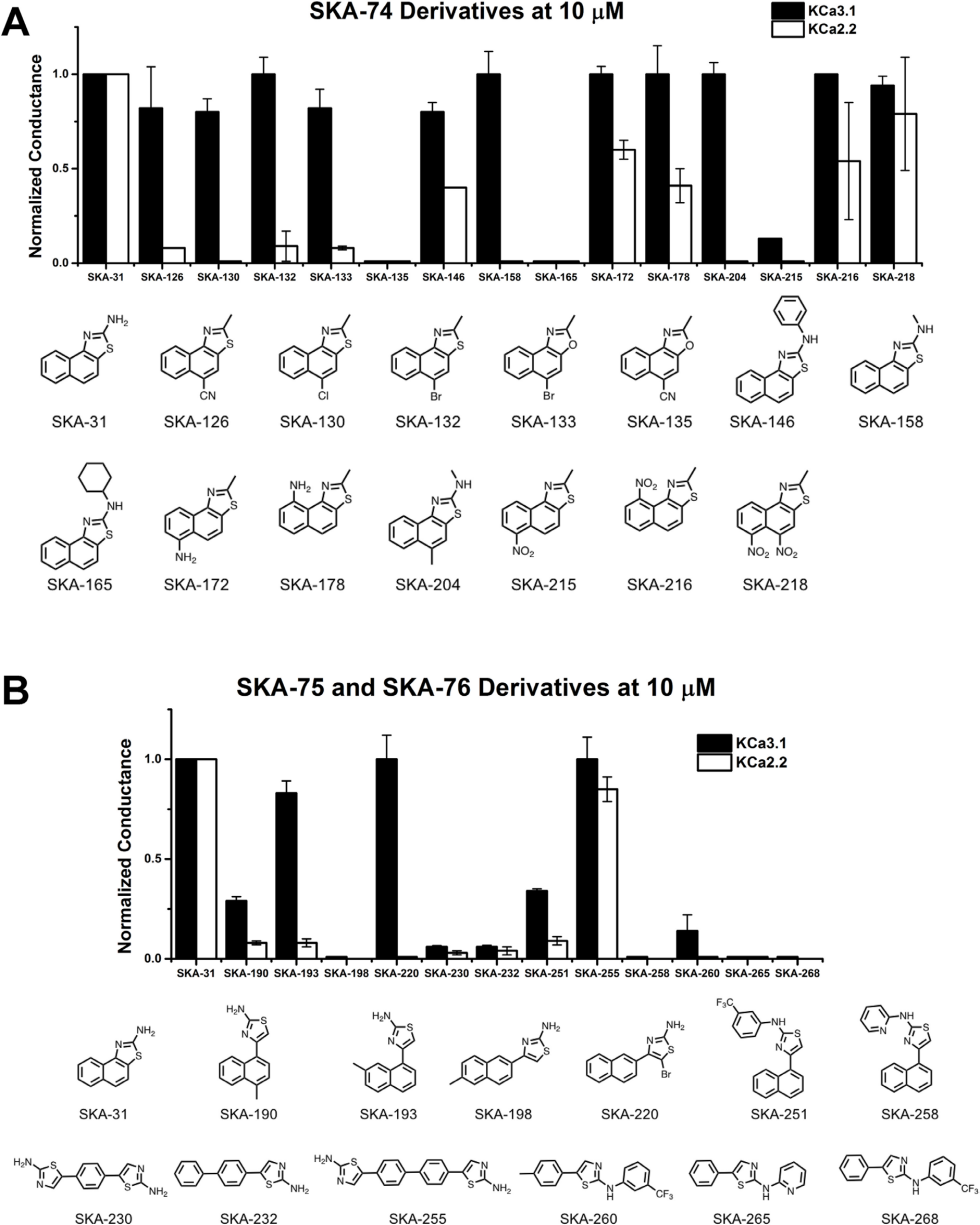
Whole-cell K_Ca_3.1 and K_Ca_2.2 responses elicited by 10 µM of activator and normalized to 10 µM of the mixed activator SKA-31. Experiments were performed by automated electrophysiology with 250 nM of free internal Ca^2+^. In each experiment the new activator was tested first, washed out with 2 saline additions, and then SKA-31 was applied as positive control for normalization of the response. **(A)** Screening of SKA-74 derivatives. **(B)** Screening of SKA-75 and SKA-76 derivatives. The bar graphs show means ± SD of the slope conductance measured between −85 and −65 mV (n = 2–7 cells). Chemical structures are shown below the bar graphs.

The newly synthesized compounds were tested by automated whole-cell patch-clamp for their ability to activate K_Ca_2.2 and K_Ca_3.1 channels stably expressed in HEK cells. In order to quickly identify promising compounds, we devised a screening system in which we measured the response of each compound at a concentration of 10 µM and normalized that response to the response elicited by 10 µM of SKA-31, which at this concentration maximally activates both K_Ca_3.1 and K_Ca_2.2 (Sankaranarayanan et al., 2009). This screen was performed at a free intracellular Ca^2+^ concentration of 250 nM, which is ideal for activator testing (Jenkins et al., 2013), and therefore allowed us to determine two things; (1) if each compound could elicit a maximal effect, and (2) if a compound displayed any selectivity towards K_Ca_2.2. A promising compound would ideally do both. **Supplementary Figure 3** shows representative raw current traces to illustrate the sequence and timing of compound additions and washes. We started by comparing the effect of the SKA-74 derivatives to SKA-31 (**Figure 4A**). Addition of a cyanide group to the C5-position (SKA-135) or of a nitro group to the 6-position (SKA-215), rendered the compounds inactive on both channels. Introduction of lipophilic chloro- or bromo-groups (SKA-130, SKA-132 and SKA-133), decreased activity on K_Ca_2.2 but preserved selectivity for K_Ca_3.1. Similarly, adding –NH_2_ or –NO_2_ groups in the 6- or 9- or both the 5- and 6-position preserved the selectivity for K_Ca_3.1 (SKA-172, SKA-178, SKA-216 and SKA-218). Substitution of the 2-position -NH_2_ group also did not result in any K_Ca_2.2 selective compounds. While introduction of a -CH_3_ group (SKA-158) abolished activity on K_Ca_2.2 but preserved it again on K_Ca_3.1, introduction of an aromatic phenyl (SKA-146) or a cyclopropyl (SKA-169) ring rendered the resulting compounds less potent than SKA-31. Introduction of a larger, aliphatic cyclohexyl group (SKA-165) completely eliminated activity on both channels.

We next tested the linear SKA-75 and SKA-76 derivatives and found that, compared to their undecorated templates, introduction of a methyl or a bromo group to SKA-75 and SKA 76 (SKA-190, SKA-193, SKA-198 and SKA-220) somewhat increased K_Ca_ channel activating potency (**Figure 4B**). However, when compared to SKA-31, none of these modifications produced the desired K_Ca_2.2 selectivity or improved potency. Additional introduction of a large substituent on the -NH_2_ group completely abolished activity (SKA-251 and SKA-258). Similarly, various linearized compounds (SKA-230, SKA-232, SKA-255, SKA-260, SKA-265 and SKA-268) were completely devoid of activity even though RosettaLigand docking predicted these linear compounds to be K_Ca_2.2 selective and potent based on multiple predicted hydrogen bonds (see **Figure 2D** for SKA-230 in K_Ca_2.2).

### Return to “Classical Medicinal” Chemistry

**Figure 5:**
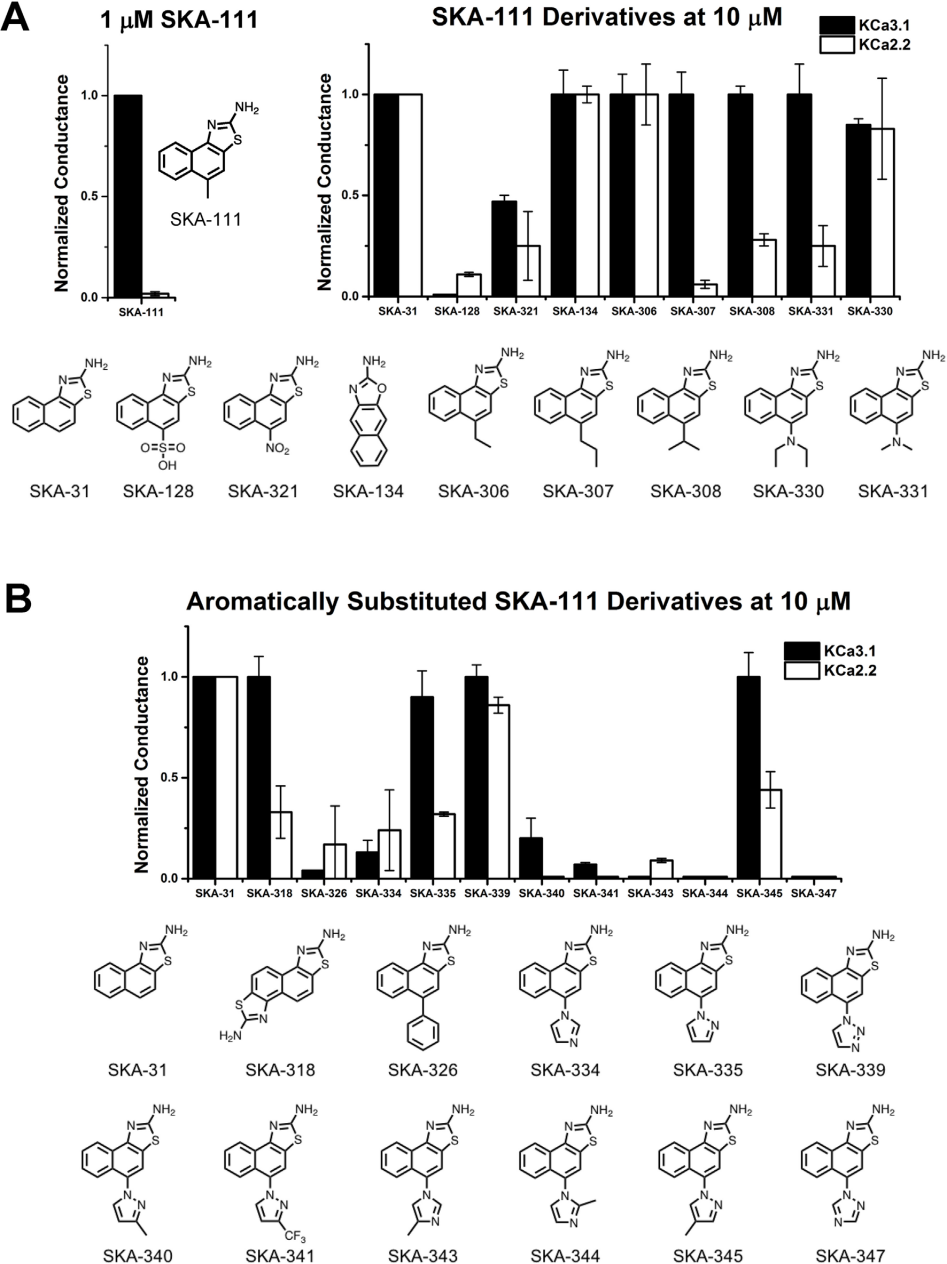
Whole-cell K_Ca_3.1 and K_Ca_2.2 responses elicited by 10 µM of activator and normalized to 10 µM of the mixed activator SKA-31. Experiments were performed by automated electrophysiology with 250 nM of free internal Ca^2+^. In each experiment the new activator was tested first, washed out with 2 saline additions, and then SKA-31 was applied as positive control for normalization of the response. **(A)** Screening of SKA-111 derivatives. **(B)** Screening of aromatically substituted SKA-111 derivatives. The bar graphs show means ± SD of the slope conductance measured between −85 and −65 mV (n = 2–7 cells). Chemical structures are shown below the bar graphs.

At this stage of our work we became very skeptical about our approach of using RosettaLigand docking for structure based K_Ca_2.2 activator design and decided to return to a purely activity driven compound design approach. We therefore went back to the 2-aminonaphothiazole system of SKA-31 and opted to further exploit our previous observation that introduction of a single -CH_3_ group in 5-position could achieve a 100-fold gain in selectivity for K_Ca_3.1 over K_Ca_2 channels (Coleman et al., 2015). In order to more thoroughly explore the structure-activity relationship (SAR) in the 5-position (**Figure 5A**) we replaced the methyl group of SKA-111 with longer alkyl chains such as ethyl (SKA-306), propyl (SKA-307), isopropyl (SKA-308) or other functional groups, which were not tried in our previous study such sulfonyl (SKA-128), nitro (SKA-321), dimethyl (SKA-330) or diethylamino (SKA-331). We further decided to generate a small library of compounds with aromatic substituents in 5-position such as phenyl, imidazole, pyrazole, and triazole in order to provide ample possibilities to pick up new van der Waals, π–π, cation–π or hydrogen bond interactions (**Figure 5B**). In total we synthesized 19 SKA-111 derivatives according to the schemes shown in **Figure 3B**. Alkyl or phenyl substituted compounds were synthesized starting from differently substituted naphthalenes, which we first nitrated using nitric acid and then reduced with hydrazine. Finally, to obtain the benzothizole ring-system the resulting amino-substituted naphthalenes were subjected to a classic Hugerschoff benzothiazole synthesis with potassium thiocyanate and liquid bromine. Secondary amines or heterocyclic ring substituted compounds were prepared from 1-fluoro-4-nitronaphthalen, which was reacted with differently substituted amines through amine arylation. This nucleophilic aromatic substitution allowed us to introduce the heterocycle ring system or alkylamine into the 5-position of the naphthalene ring. Other 2-aminobenzothiazoles were generated through nitration and Hugerschoff benzothiazole synthesis.

We next proceeded to screen these SKA-111 derivatives (**Figure 5**) and found that replacement of the 5-position methyl group with ethyl (SKA-306), propyl (SKA-307), isopropyl (SKA-308) and dimethylamine (SKA-331) resulted in compounds that were roughly as potent and as K_Ca_3.1 selective as SKA-111. Addition of a diethylamine group (SKA-330) somewhat reduced potency, while large, polar substituents like a nitro-group (SKA-312) or a sulfonic acid (SKA-128) drastically reduced potency on both K_Ca_3.1 and K_Ca_2.2. Unfortunately, introduction of aromatic substituents in 5-positions also did not provide any K_Ca_2.2 selective compounds and overall again reduced potency on both channels. Taken together, we failed to generate any K_Ca_2.2 selective SKA compounds using either structure based or “classical” approaches.

### The Full-Length K_Ca_3.1 Cryo-EM Structure Reveals That the C-Terminal CaM-BD/CaM Dimer Crystal Is an Artefact

In our structure-based drug design attempt described above, we had used the C-terminal CaM-BD/CaM crystal structure, which consist of two vertically orientated CaM molecules and two horizontal K_Ca_2.2 C-terminal fragments in an antiparallel arrangement (Zhang et al., 2012). In this structure, the CaM N-lobe interacts with the C-terminal region of the CaM-BD, whereas the C-lobe is bound to N-terminal region of the CaM-BD. Several K_Ca_ channel activators, EBIO (Zhang et al., 2012), NS309 (Zhang et al., 2013) and, most recently in a publication from the Structural Biology group at Pfizer (Cho et al., 2018), CyPPA and riluzole were shown by X-ray crystallography and solid-state NMR to be located at the interface between the CaM N-lobe and the C-terminal region of the CaM-BD, where we docked our compounds. For clarity, only half of this so-called “dimer of dimers” complex, one CaM and one C-terminal fragment, was used for our modeling (see **Supplementary Figure 1**).

**Figure 6:**
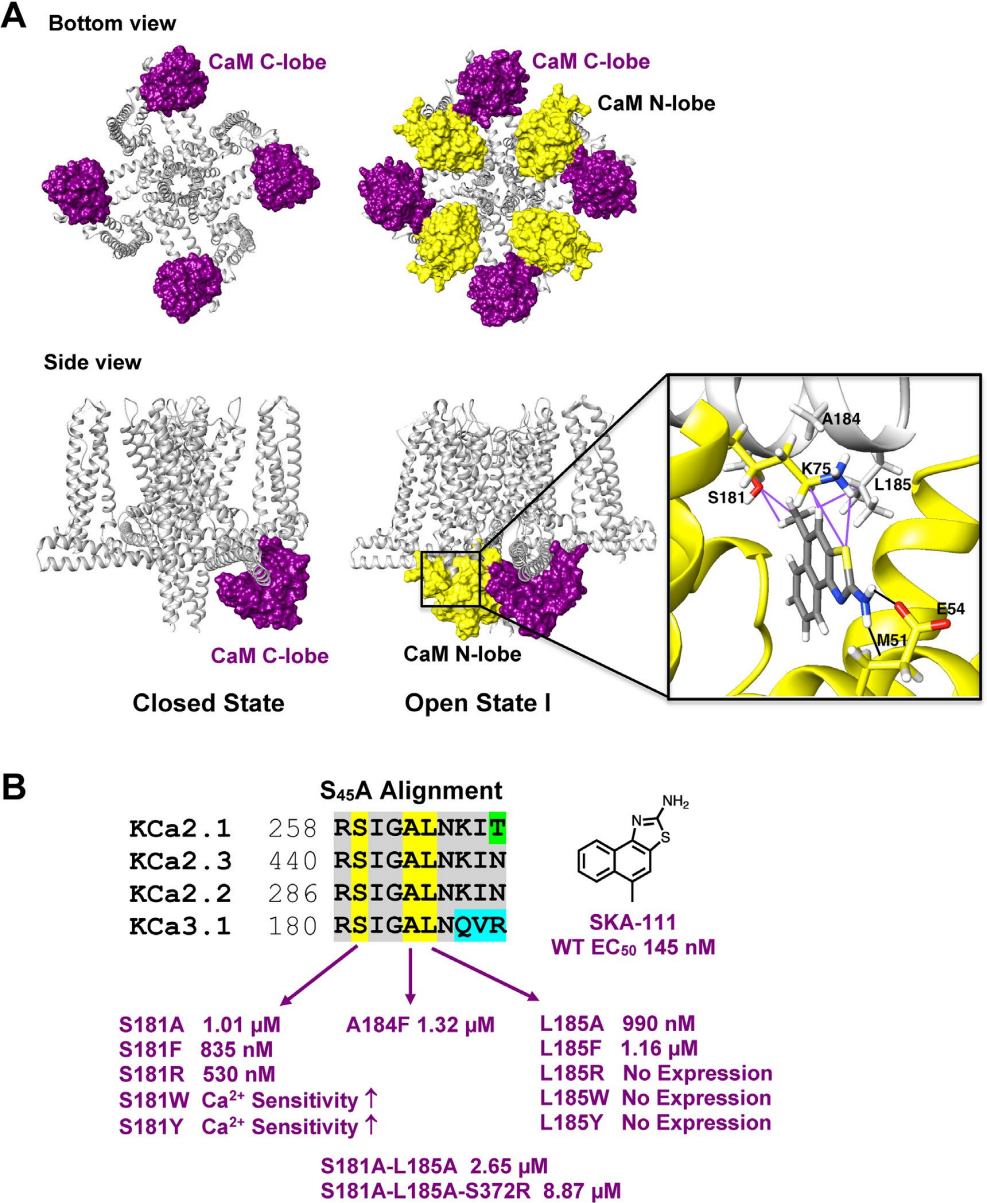
Docking model of SKA-111 in the full-length K_Ca_3.1 structure and mutational strategy. **(A)** Bottom and side view of the full-length K_Ca_3.1 cryo-EM structure following Rosetta refinement in the Ca^2+^ free closed state (pdb: 6cnm) and Open state I (pdb: 6cnn). The channel is shown in gray, the CaM C-lobe in purple and the CaM N-lobe in yellow. Next to open state 1 we show a zoom out of the lowest energy docking pose of SKA-111 in the interface between the CaM N-lobe and the S45A helix interface. Hydrogen bonds are shown in black, van der Waals interactions are visualized in purple. For clarity, not all side chains of CaM residues within contact range of SKA-111 are explicitly shown. Please note the channel residues S181, A184 and L185. **(B)** Alignment of the S_45_A helix sequence in K_Ca_2.1, K_Ca_2.2, K_Ca_2.3 and K_Ca_3.1. Residues that were mutated are highlighted in yellow and the EC_50_ values for SKA-111 shown next to each mutant (for confidence intervals see **Figures 7** and **8**).

Recently, the MacKinnon group (Lee and MacKinnon, 2018) determined the full-length cryo-EM structures of K_Ca_3.1 in the closed and in two activated states (pdb: 6cnm, 6cnn, and 6cno) and revealed that the C-terminal CaM-BD/CaM dimer crystal is an artefact. The full-length structure showed four CaMs per channel tetramer, with the CaM C-lobe of each CaM tightly bound to the CaM-BD of each subunit in the closed and the two activated states (**Figure 6A**). However, the N-lobes were only clearly visible in the open, Ca^2+^-bound states and poorly resolved in the closed, Ca^2+^-free structure suggesting that they are flexible in the absence of Ca^2+^. When Ca^2+^ binds to the N-lobe it moves from the bottom of the S2 segment to the bottom of the S4-S5 linker (which in K_Ca_3.1 consists of two helices), while the C-lobe maintains its interaction with the HA and HB helices in the C-terminus. The N-lobe then pulls part of the S4–S5 linker, namely the S_45_A helix downward and this displacement expands the S6 helices and opens the pore (Lee and MacKinnon, 2018). In their study the MacKinnon group also proposed a new binding pocket for the K_Ca_ activator EBIO formed by the S_45_A helix and the CaM N-lobe, in which EBIO binds to L185 in the S_45_A linker (Lee and MacKinnon, 2018) instead of L480 in the C-terminal crystal complex (Zhang et al., 2012). However, this very plausible alternative binding site hypothesis was not experimentally tested.

### Probing the “New” SKA Compound Binding Site at the Interface Between the S_45_A Helix and the CaM N-Lobe

Based on this new binding site hypothesis we here docked and energy minimized SKA-111 in the interface pocket between the S_45_A helix and the CaM N-lobe (**Figure 6A**) using the open state of the full length cryo-EM structure of K_Ca_3.1. The two open cryo-EM structures (6cnn and 6cno) were refined using the Rosetta cryo-EM refinement protocol with cryo-EM density map (Wang et al., 2016). A total of 10,000 models were generated for each state and the model with the lowest energy among the largest clusters of the top 1,000 models was used for RosettaLigand docking of SKA-111 starting from random ligand positions at the interface between the S_45_A helix and the CaM N-lobe. Similar to our previous docking model, SKA-111 is stabilized in open state 1 by hydrogen bonds involving M51 and E54 and multiple CaM N-lobe residues are located within a 5 Å radius sphere around SKA-111 (F19, I27, L32, M51, I52, E54 V55, I63, F68, M71, M72, R74, K75) (**Figure 6A**, open state 1). However, on the channel side SKA-111 now makes van der Waals interactions with S181 and L185 in the S_45_A helix (**Figure 6A**). Interestingly, in open state 2 the lowest energy docking pose of SKA-111 is “flipped” 180 degrees around the hydrogen bond with CaM M51 and the molecule now makes van der Waals contacts with S181 and A184 in the S_45_A helix and again multiple residues in the CaM N-lobe (**Supplementary Figure 4**). The parent compound SKA-31 and the benzoxazole SKA-121 take up similar low energy docking poses in which they make contacts with M51 and E54 in CAM and S181 and/or L185 in K_Ca_3.1 (**Supplementary Figure 5**).

**Figure 7:**
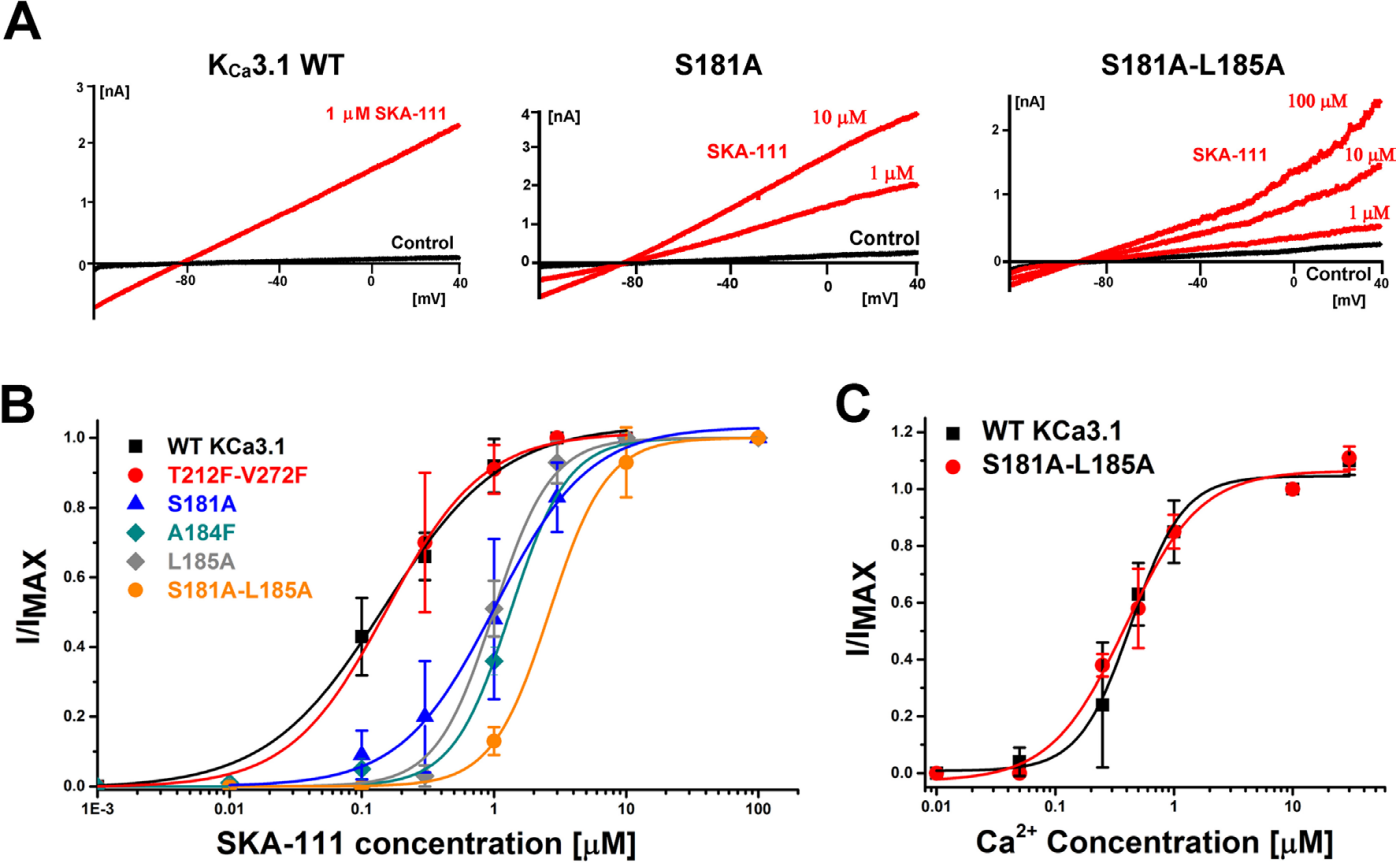
Mutations of S181 and L185 in the S_45_A helix disturb SKA-111 activity but not calcium gating. **(A)** Representative whole-cell WT and mutant K_Ca_3.1 currents with an intracellular free calcium concentration of 250 nM in the presence and absence of SKA-111. **(B)** Concentration–response for SKA-111 induced current activation: WT (EC_50_ = 146 nM, 95% CI: 99–193 nM), T212F-V272F (EC_50_ = 153 nM, 95% CI: 114–182 nM, P = 0.1017), S181A (EC_50_ = 1.012 µM, 95% CI: 0.780–1.244 µM, P < 0.0001), A184F (EC_50_ = 1.326 µM, 95% CI: 1.205–1.447 µM, P < 0.0001), L185A (EC_50_ = 0.993 µM, 95% CI: 0.903–1.083 µM, P < 0.0001), S181A-L185A (EC_50_ = 2.654 µM, 95% CI: 2.619–2.624 µM, P < 0.0001). Whole-cell K_Ca_3.1 currents were elicited by voltage-ramps from −120 to + 40 mV with an intracellular free calcium concentration of 250 nM. Data points are mean ± S.D. from 3–5 independent cells/recordings. The reported P values are for an extra sum-of-squares F test (GraphPad Prism5; GraphPad Software, La Jolla, CA) to compare the curves of K_Ca_3.1 mutants to WT. **(C)** Inside-out calcium concentration-response curves for WT K_Ca_3.1 (EC_50_ = 437 nM, 95% CI: 353–521 nM, n*_H_* = 1.98) and the S181A-L185A double mutant (EC_50_ = 392 nM, 95% CI: 287-497 nM, n*_H_* = 1.39, P = 0.4951). Data points are the mean ± S.D. from 3–5 independent recordings. The calcium-sensitivity of the mutant is statistically not different from the WT K_Ca_3.1 channel (P = 0.4951 in extra sum-of-squares F test).

In order to probe this “new” binding site we decided to mutate S181, L185 and the neighboring A184 to smaller, bulkier or charged residues to either disrupt contacts with SKA-111 or disturb the overall shape and size of the S_45_A helix/CaM N-lobe interface pocket. As described in the *Materials and Methods*, mutations were first tested for expression and Ca^2+^ sensitivity and then used for evaluation of SKA-111 sensitivity. Mutating serine 181 to a shorter alanine, reduced SKA-111 potency 7-fold by removing the van der Waals contact to the 5-position –CH_3_ group of SKA-111 (**Figures 6B** and **7**). Mutating S181 to a charged arginine or large phenylalanine also reduced SKA-111 potency presumably by “pushing” SKA-111 forward. Introducing even larger tryptophan or tyrosine residues had an interesting effect on K_Ca_3.1 gating and resulted in channels that already produced large nA currents in the presence of only 250 nM free Ca^2+^, suggesting that these mutants are more sensitive to Ca^2+^ then the wild-type channel. We therefore did not use these two mutants for testing SKA-111 sensitivity. Mutating the other S_45_A helix residue, L185, that is in direct van der Waals contact with SKA-111 had a similar effect as mutating position 181. Replacing leucine 185 with a smaller alanine reduced SKA-111 potency by 7-fold, while substitution of a larger phenylalanine in 185-position produced an 8-fold reduction in potency (**Figures 6B** and **7**). Introduction of even larger (W, Y) or charged residues unfortunately resulted in non-functional K_Ca_3.1 channels that no longer respond to free Ca^2+^ concentrations as high as 10 µM (**Figure 6B**). Although it is not in direct contact with SKA-111 we also mutated the neighboring A184 position to F because our model suggested that a phenylalanine in this position would push the CaM-K75 residue, that is contacting SKA-111 upwards. In keeping with this hypothesis, the A184F mutant was 9-fold less sensitive to SKA-111 than the WT K_Ca_3.1 channel (**Figures 6B** and **7**).

In order to more dramatically reduce SKA-111 sensitivity we next generated the double S181A-L185A mutant reasoning that it would disrupt both the van der Waals interactions SKA-111 makes with the S_45_A helix. In keeping with this idea, the S181A-L185A mutant produced a roughly additive effect when compare with the two single A mutations and reduced SKA-111 potency by 18-fold (**Figures 6B** and **7**). In order to determine that this change in SKA-111 sensitivity is not caused by a reduced Ca^2+^ sensitivity we performed in-side out recordings comparing the Ca^2+^ sensitivity of the S181A-L185A mutant with the WT K_Ca_3.1 channel and found that mutant did not differ in its Ca^2+^ sensitivity (**Figure 7C**). As a further control experiment, we tested the sensitivity of a double “fenestration” mutation, T212F-V272F, to SKA-111. We had previously generated this mutation, which closes the K_Ca_3.1 fenestration between S5 and S6 with two bulky aromatic residues without changing the biophysical properties of the channel when identifying the binding site of the dihydropyridine nifedipine in K_Ca_3.1 (Nguyen et al., 2017). As expected, the double “fenestration” mutant was as sensitive to SKA-111 as the WT channel (**Figure 7**).

### Why Did the S372R Mutation in Our Previous Study Have Such a Large Effect of K_Ca_ Activator Potency?

**Figure 8:**
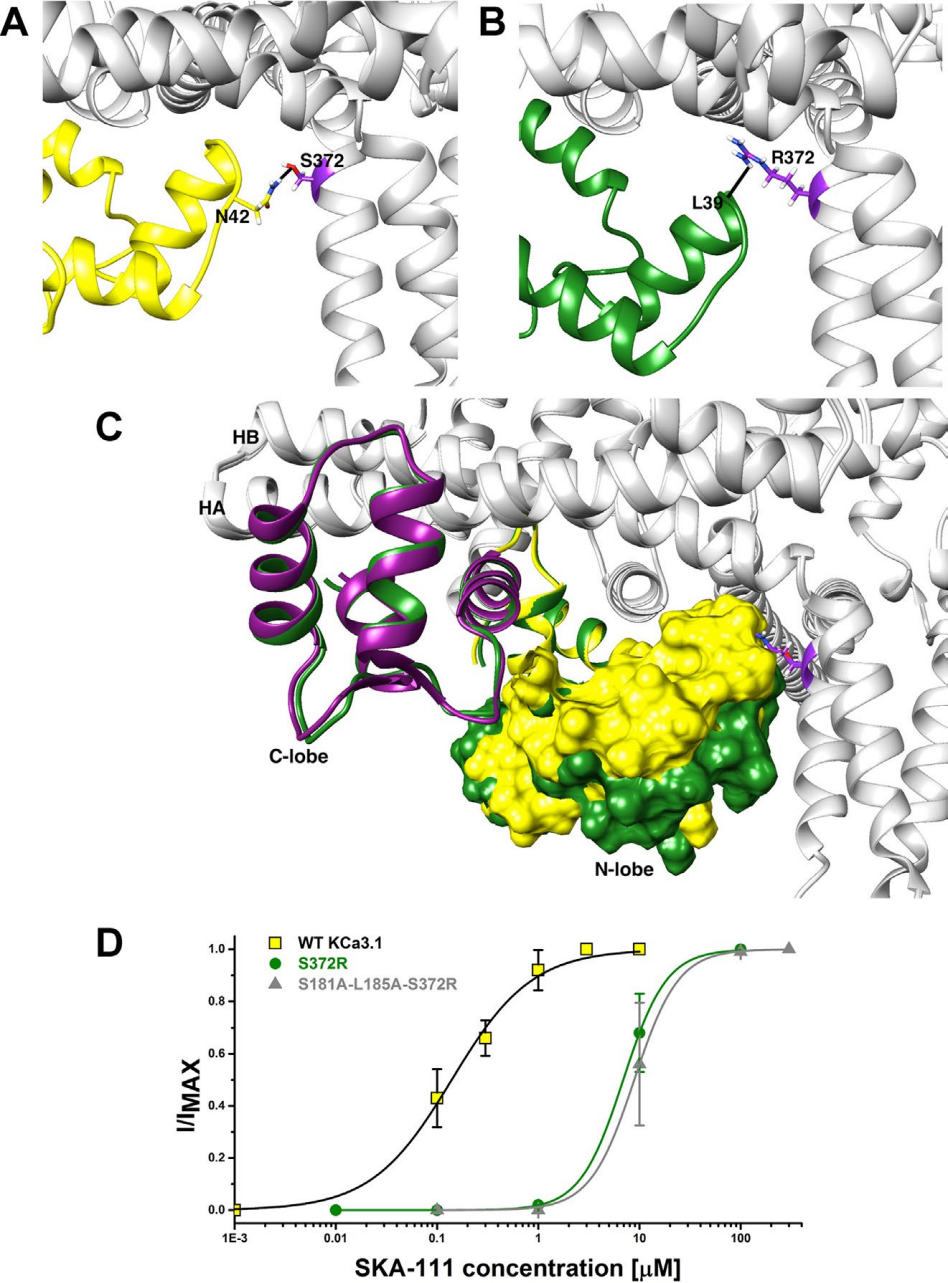
Rosetta Model of the wild type K_Ca_3.1 channel in open state 1 with serine in position 372 **(A)** and of the S372R mutant **(B)**. Hydrogen bonds are visualized in black. **(C)** Overlay of the Rosetta models of the S_45_A helix/CaM N-lobe interface pocket in the wild type (yellow) and the S372R mutant shown in space fill. **(D)** Concentration-response curve for SKA-111 induced current activation: WT (EC_50_ = 146 nM, 95% CI: 99–193 nM), S372R (EC_50_ = 6.860 µM, 95% CI: 6.788–6.932 µM, P < 0.0001), S181A-L185A-S372R (EC_50_ = 8.876 µM, 95% CI: 8.652–9.100 µM, P < 0.0001). Data points are mean ± S.D. from 3–5 independent cells/recordings.

Taken together the above presented mutagenesis data suggest that the K_Ca_ channel activator SKA-111 is indeed binding in the interface between the S_45_A helix and the CaM N-lobe as hypothesized by Lee et al. (Lee and MacKinnon, 2018). However, we were still puzzled by the fact that we had previously seen such a strong reduction in potency for multiple K_Ca_ channel activators including SKA-31, SKA-111, EBIO and NS309, when mutating S372 in K_Ca_3.1 or the corresponding S632 residue in K_Ca_2.3 to arginine (Brown et al., 2017). Based on the C-terminal crystal fragment we had believed this residue to be located at the back of the interfacial binding site pocket. In the full-length K_Ca_3.1 structure S372 is located at the top of the C-terminal HC helix, which forms a coiled coil located at the center of the channel. As shown in **Figure 8A**, S372 is facing outward from this coil towards N42 in the CaM N-lobe and accepting a hydrogen bond from its NH_2_ group. Mutating S372 to a larger, charged arginine (**Figure 8B**) in our Rosetta model of open state I “pushes” the outer edge of the CaM N-lobe 6.4 Å downwards, now resulting in a hydrogen bond with L39 in the CaM N-lobe and changing the shape and the volume of the interface pocket (see **Figure 8C** for an overlay of the WT and mutant channel). In keeping with this “distortion” of the interface pocket SKA-111 does not converge in the S372R mutant, while all top 50 lowest energy-binding poses virtually overlay in the WT channel pocket (**Supplementary Figure 6**). Interestingly, introducing the double AA mutation (S181A-L185A) into the S372R mutant, resulted only in small, but not statistically significant additional shift in the concentration-response curve of SKA-111 (**Figure 8D**).

## Discussion

We here attempted to perform structure assisted design of K_Ca_2.2 selective small molecule activators using the K_Ca_2.2 CaM-BD/CaM crystal structure. This structure seemed extremely attractive for a structure based approach because it had been repeatedly crystalized by multiple groups. The first structure of the K_Ca_2.2 channel C-terminal calmodulin binding domain (CaM-BD) in complex with CaM was reported in 2001 at 1.6 Å resolution and showed an elongated, anti-parallel dimer of two K_Ca_2.2 C-terminal 76 amino acid long fragments. On each of these channel pieces CaM was tightly bound with its C-lobe to two alpha helices connected by a turn from the same channel subunit and “grabbed” the free end of the fragment from the other subunit in the dimer with its N-lobe suggesting that CaM-BD dimerization might gate K_Ca_2 channels (Schumacher et al., 2001). The same dimeric crystal orientation was afterwards repeatedly observed in structural studies addressing the mechanism of action of small molecule K_Ca_ channel activators and of PIP_2_ on K_Ca_2.2 channel function (Zhang et al., 2012; Zhang et al., 2013; Zhang et al., 2014). Zhang et al. showed in several high-resolution X-ray structures that the K_Ca_ activator EBIO, the more potent NS309, as well as several NS309 derivatives bind in the interface between the CaM N-lobe and the K_Ca_2.2 CaM-BD (Zhang et al., 2012; Zhang et al., 2013; Nam et al., 2017). Independently of this work a group at Pfizer also crystallized the K_Ca_2.2 CaM-BD/CaM complex and showed the presence of two other K_Ca_ activators, riluzole and a CyPPA derivatives, in the interface between the CaM N-lobe and the channel CaM-BD (Cho et al., 2018). This interaction was further confirmed by solution state NMR experiments demonstrating that riluzole and the CyPPA analog perturb the chemical shifts for both ^15^N-labeled CaM and ^15^N-labeled K_Ca_2.2 CaM-BD fragments (Cho et al., 2018). Moreover, our own previous molecular modeling studies using the K_Ca_2.2 CaM-BD/CaM crystal structure to generate a homology model of the analogous binding pocket for the related K_Ca_3.1 channel in order to explain the K_Ca_3.1 selectivity of our K_Ca_ activators SKA-111 and SKA-121 (Coleman et al., 2014), seemed in good agreement with experimental mutagenesis and structure-activity relationship observations for the SKA-type activators (Brown et al., 2017). As described above we were therefore very astonished when our computationally designed SKA molecules, which based on the Rosetta modeling work should have been both K_Ca_2.2 selective and potent, showed such disappointing activity in electrophysiological experiments. By demonstrating that the C-terminal CaM-BD/CaM crystal is an artefact the full-length K_Ca_3.1 structure (Lee and MacKinnon, 2018) explained this conundrum for us. The study also provided us with a very testable structural hypothesis for an alternative K_Ca_ activator binding site in between the highly flexible CaM N-lobe and the S_45_A helix in the unusually long S4-S5 linker of K_Ca_3.1.

**Figure 9:**
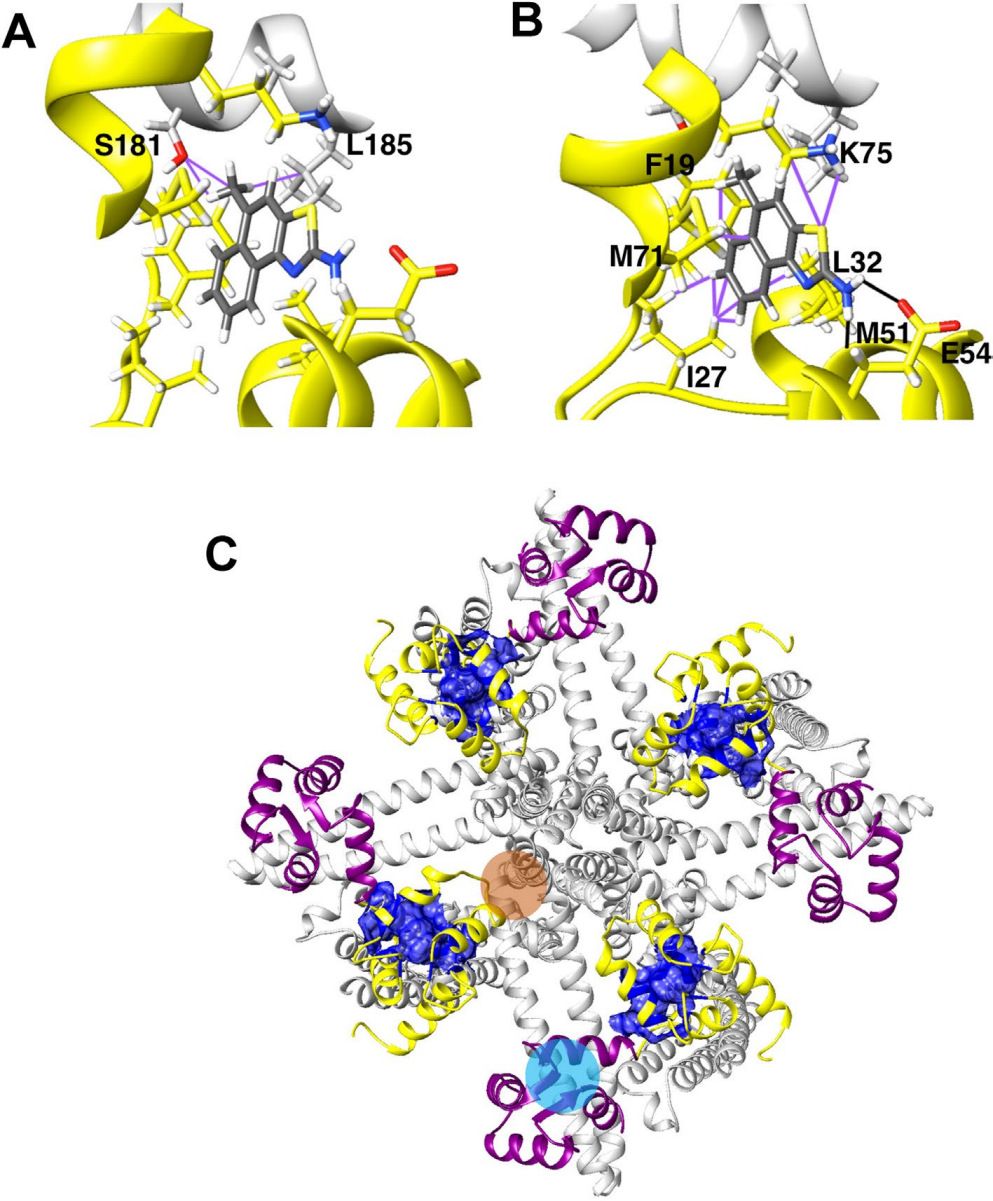
Interactions between SKA-111 and residues in the K_Ca_3.1 S_45_A helix **(A)** and the CaM N-lobe **(B)** interface pocket. Hydrogen bonds are indicated by black lines, van der Waals interactions are indicated by purple lines. **(C)** The K_Ca_3.1-CaM channel complex seen from the intracellular side. The S_45_A helix/CaM N-lobe interface colored dark blue. Two other, potential binding sites for small molecules are highlighted with orange or sky-blue circles. The channel is shown in gray, the CaM C-lobe in purple and the CaM N-lobe in yellow.

We here tested this hypothesis from the MacKinnon laboratory and found that at least our K_Ca_ channel activator SKA-111 is indeed binding in the interface pocket between the S_45_A helix and the CaM N-lobe. Interestingly, while the channel residues implicated in SKA-111 binding, namely S181, A184 and L185 (**Figure 9A**), are now different, the residues in the CaM N-lobe (F19, I27, L32, M51, E54, M71, and K75) are the same residues as in our previous binding pocket (**Figure 9B** and **Supplementary Figure 1**). In both the “old” and the new binding pocket the amino group of the benzothiazole ring of SKA-111 forms two hydrogen bonds with M51 and E54 in the CaM N-lobe and makes nine van der Waals contacts with additional CaM residues. We suspect that these multiple, strong interactions with CaM are responsible for the fact that the benzothiazole riluzole and the structurally related EBIO and NS309 could be soaked into the C-terminal crystal where the highly flexible N-lobe of CaM had “grabbed” the only available part of the C-terminal helix that was not already occupied by the C-lobe. And as chance would have it the sequence in this part of the K_Ca_2.2 C-terminus also contains a serine and leucin, similarly spaced apart, creating a very similar environment on the channel side for additional van der Waals contacts. The fact, that the majority of the contacts that SKA-111 is making in the interface pocket between the S_45_A helix and the CaM N-lobe are with CaM are also in line with our findings that we could significantly alter SKA-111 potency by mutating K_Ca_3.1 residues in the S_45_A helix but not render K_Ca_3.1 completely insensitive to its activating activity without rendering the channel non-functional through more drastic mutations.

We believe we have now identified the “correct” binding site for SKA-111 and related SKA compounds. However, since we only used mutagenesis in combination with functional electrophysiological recordings to confirm the binding site, there remains the caveat that we could have observed allosteric effects. It will therefore be important to perform additional experiments in future that demonstrate binding to the S_45_A helix for example with a photo-affinity probe and to directly measure ligand binding affinities using surface plasma resonance, isothermal titration calorimetry or differential scanning fluorimetry. These experiments should be performed with physiological ligand concentrations and not with saturating concentrations as was the case in the above described crystallography and solution state NMR studies in order to avoid the identification of “non-physiological relevant” binding sites.

Based on the K_Ca_3.1 structural model we would further like to hypothesize here that the cytoplasmic facing surface of K_Ca_3.1 and the closely related K_Ca_2 channels offer multiple binding pockets for positive and potentially also negative gating modulators. As visualized in **Figure 9C** in dark blue, the S_45_A helix/CaM N-lobe interface is present four times in the complex consisting of four K_Ca_3.1 α-subunits and four CaM molecules. How many of these pockets can be occupied by a small molecule activator is currently unknown and we would like to posit that it is possible that the occupancy of these four pockets might differ between different activators molecules and different K_Ca _channel subtypes. In addition to the S_45_A helix/CaM N-lobe interface, the SiteMap function of the Schrödinger Glide software identifies two other potential druggable sites in the cytoplasmic surface of K_Ca_3.1 that could accommodate small molecules: one adjacent site located between the S_45_B helix and the HA helix in the C-terminus (orange circle in **Figure 9C**) and another site in the space between the CaM C-lobe and the HB helix in the C-terminus (sky blue circle in **Figure 9C**). Additional experimental and structural work will be necessary to determine if any, and how many, of these sites are targeted by positive or negative K_Ca_ channel gating modulators.

## Data Availability

The physical data of all synthesized compounds are provided in the Method section of this article. NMR spectra are available on request. Protein Data Bank (pdb) format files of the Rosetta models of K_Ca_3.1 open state 1 and open state 2 with SKA-111 docked in the interface between S_45_A helix and the CaM N-lobe are provided in **footnote 1**; pdb files of all other models are available upon request.

## Author Contributions

Participated in research design: HS, BB, VY-Y, HW. Performed chemical synthesis and compound analysis: HS, LS, VS. Conducted patch-clamp recordings and data analysis: BB. Performed molecular modeling and related data analysis: HS, VY-Y. Determined crystal structures: JF. Wrote or contributed to the writing of the manuscript: BB, HS, VY-Y, HW.

## Funding

This work was supported by the CounterACT Program, National Institutes of Health Office of the Director [U54NS079202], and the National Institute of Neurological Disorders and Stroke [R21NS101876]. BB was supported by the National Center for Advancing Translational Sciences, National Institutes of Health, through grant number UL1 TR001860 and linked award TL1 TR001861.

## Conflict of Interest Statement

The authors declare that the research was conducted in the absence of any commercial or financial relationship that could be constructed as a potential conflict of interest.

## Footnotes

1
https://frontiersin.figshare.com/collections/The_Trials_and_Tribulations_of_Structure_Assisted_Design_of_KCa_channel_Activators/4641455
