# Supplementary material for: The Trials and Tribulations of Structure Assisted Design of KCa Channel Activators
Source: Front Pharmacol. 2019 Sep 20;10:972. doi: 10.3389/fphar.2019.00972 (PMC6764326; doi:10.3389/fphar.2019.00972)
Supplement: Supplementary file 1 [file DataSheet_1.pdf]

Supplementary Fig.1

SKA-111 in $K_{Ca}3.1$ **SKA-111 in $K_{Ca}2.2$**

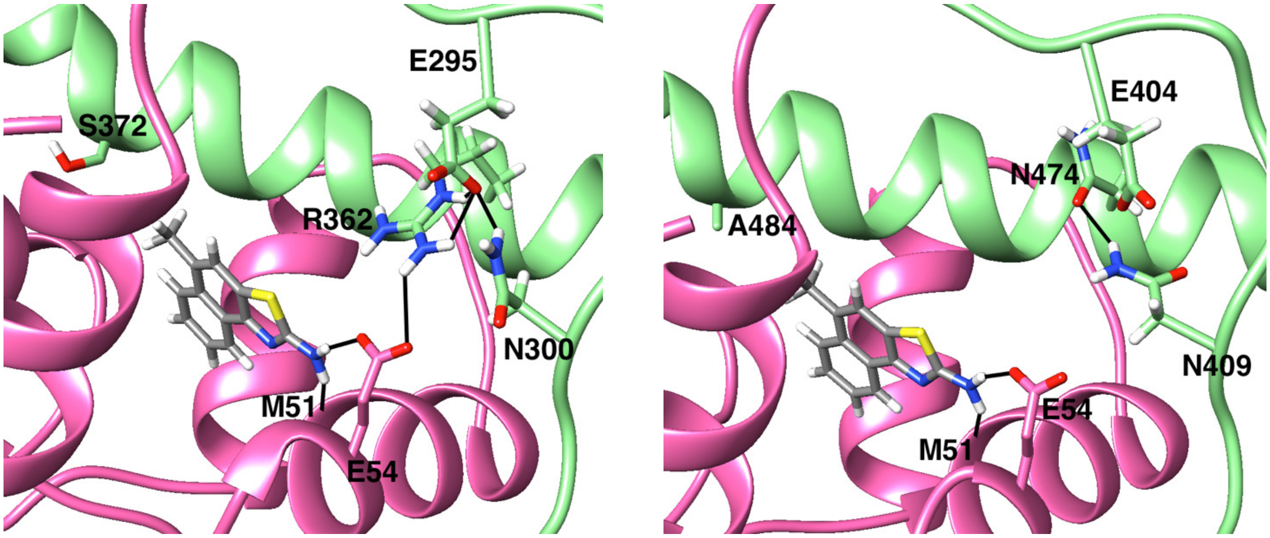

Docking model of $K_{Ca}3.1$ and $K_{Ca}2.2$ CaM-BD/CaM complexes with SKA-111. In the $K_{Ca}3.1$ model the amino group of the benzothiazole ring system of SKA-111 forms hydrogen bonds with M51 and E54 in calmodulin. E54 is further stabilized by an extensive hydrogen bond network with R362, E295 and N300 in the $K_{Ca}3.1$ channel model, which we hypothesized to be responsible for the 100-fold $K_{Ca}3.1$ selectivity of SKA-111. In the $K_{Ca}2.2$ model, however, SKA-111 forms only hydrogen bonds with M51 and E54. Due to the shorter length of the N474 side chain in $K_{Ca}2.2$ E54 is not stabilized by additional hydrogen bonds. The CaM-BD is shown in light green; CaM is colored pink.

The eight 2-aminobenzothiazoles shown below, which all display some degree of selectivity for $K_{Ca}3.1$ over $K_{Ca}2$ channels, converged on similar binding poses as SKA-111 with an extensive hydrogen bond network centered around R362 in the $K_{Ca}3.1$ model and hydrogen bonds between the $-NH_2$ group and M51 and E54 in CaM in both the $K_{Ca}3.1$ and the $K_{Ca}2.2$ model.

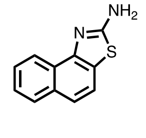	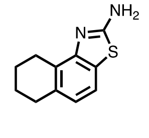	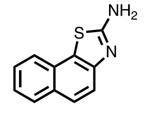	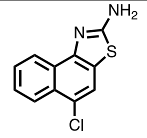	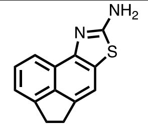	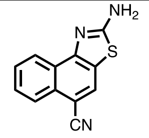	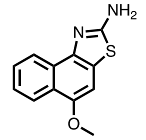	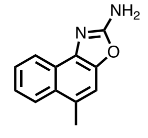
SKA-31	SKA-44	SKA-45	SKA-72	SKA-73	SKA-107	SKA-117	SKA-120

Supplementary Fig.2

Chemical structures of the 168 virtually designed and docked compounds.
Highlighted compounds were synthesized and tested.

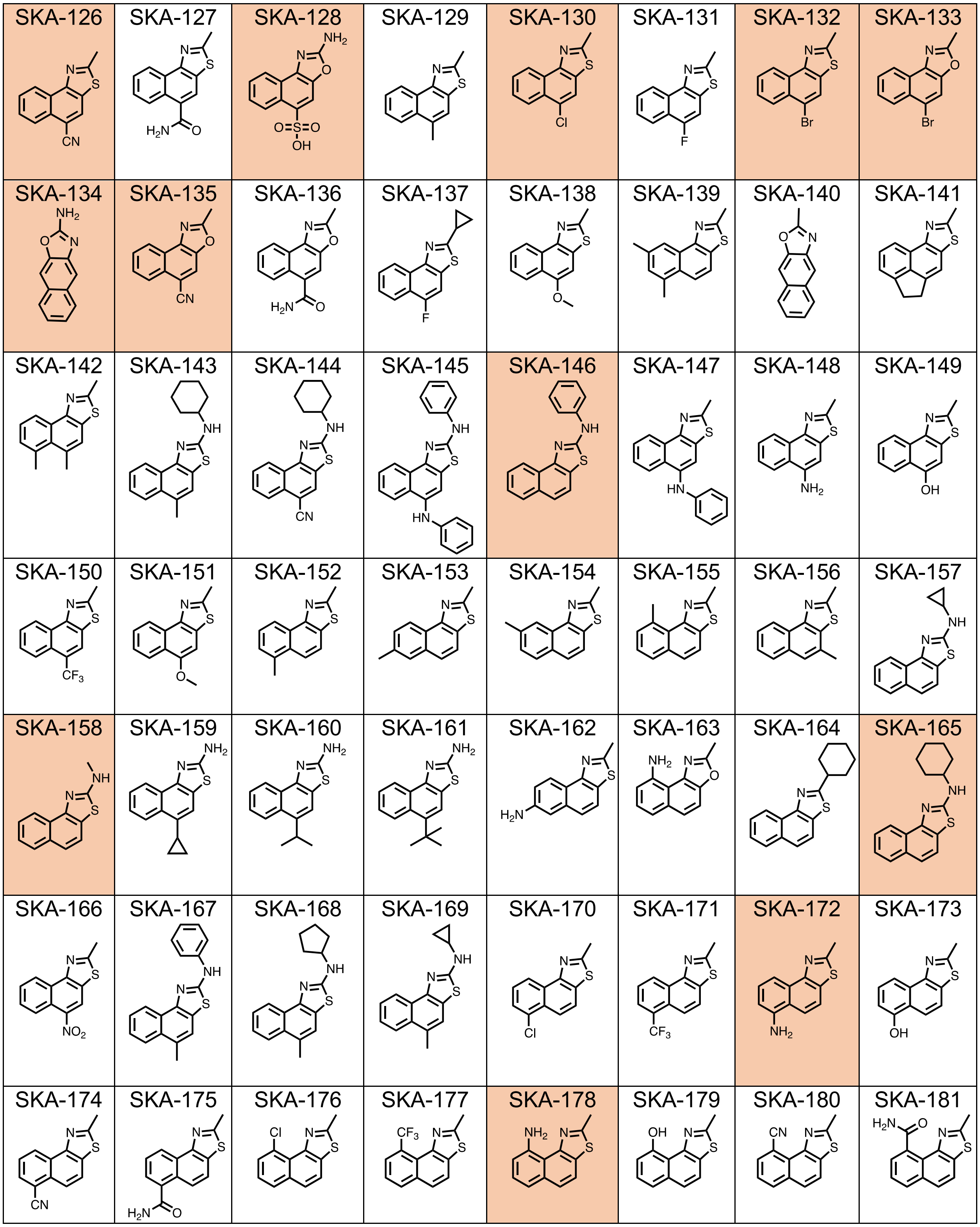

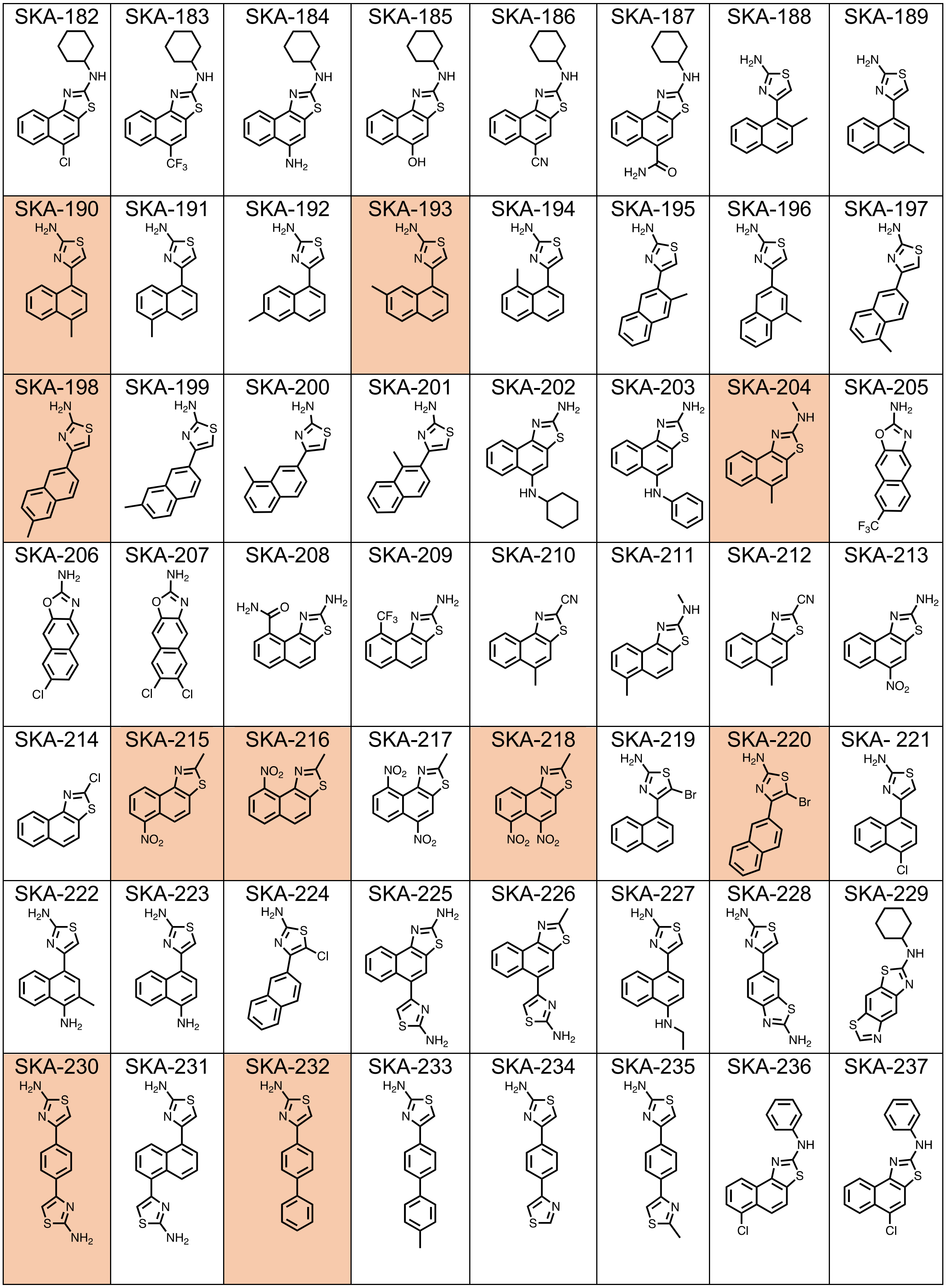
SKA-182 SKA-183 SKA-184 SKA-185 SKA-186 SKA-187 SKA-188 SKA-189
SKA-190 SKA-191 SKA-192 SKA-193 SKA-194 SKA-195 SKA-196 SKA-197
SKA-198 SKA-199 SKA-200 SKA-201 SKA-202 SKA-203 SKA-204 SKA-205
SKA-206 SKA-207 SKA-208 SKA-209 SKA-210 SKA-211 SKA-212 SKA-213
SKA-214 SKA-215 SKA-216 SKA-217 SKA-218 SKA-219 SKA-220 SKA- 221
SKA-222 SKA-223 SKA-224 SKA-225 SKA-226 SKA-227 SKA-228 SKA-229
SKA-230 SKA-231 SKA-232 SKA-233 SKA-234 SKA-235 SKA-236 SKA-237

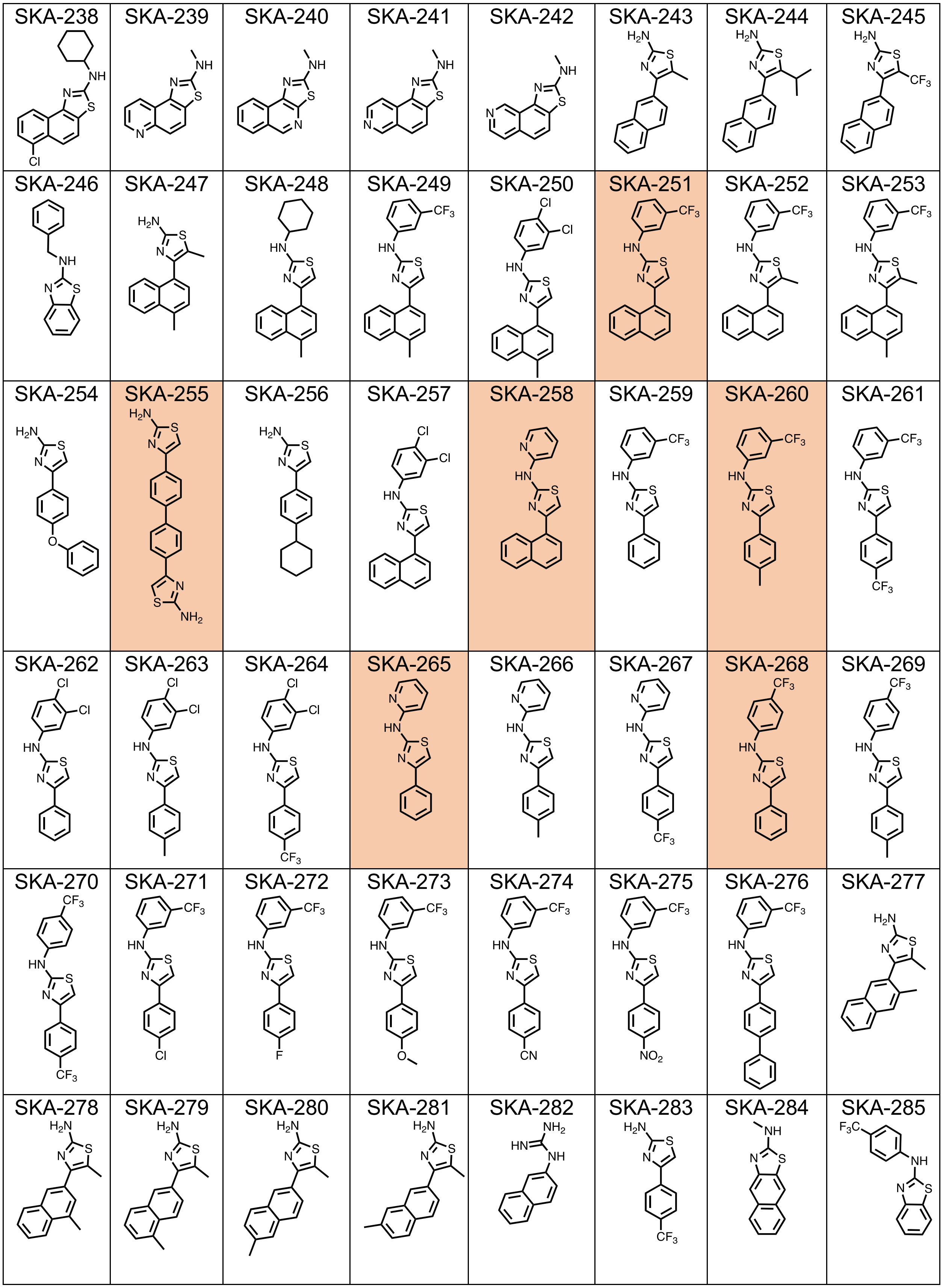
SKA-238 SKA-239 SKA-240 SKA-241 SKA-242 SKA-243 SKA-244 SKA-245
SKA-246 SKA-247 SKA-248 SKA-249 SKA-250 SKA-251 SKA-252 SKA-253
SKA-254 SKA-255 SKA-256 SKA-257 SKA-258 SKA-259 SKA-260 SKA-261
SKA-262 SKA-263 SKA-264 SKA-265 SKA-266 SKA-267 SKA-268 SKA-269
SKA-270 SKA-271 SKA-272 SKA-273 SKA-274 SKA-275 SKA-276 SKA-277
SKA-278 SKA-279 SKA-280 SKA-281 SKA-282 SKA-283 SKA-284 SKA-285

SKA-286

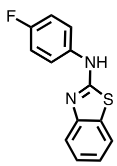

SKA-287

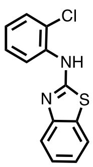

SKA-288

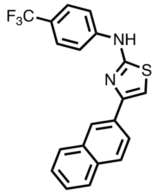

SKA-289

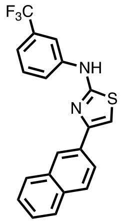

SKA-290

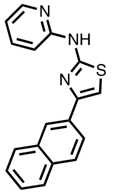

SKA-291

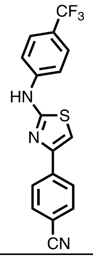

SKA-292

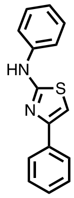

SKA-293

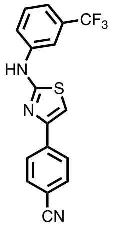

Supplementary Fig.3

QPatch screening of activator compounds on $K_{Ca}3.1$ and $K_{Ca}2.2$ channels with 250 nM of free Ca^{2+} in the internal solution.

Screening of SKA-126 on $K_{Ca}3.1$

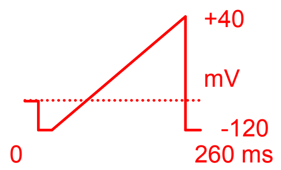

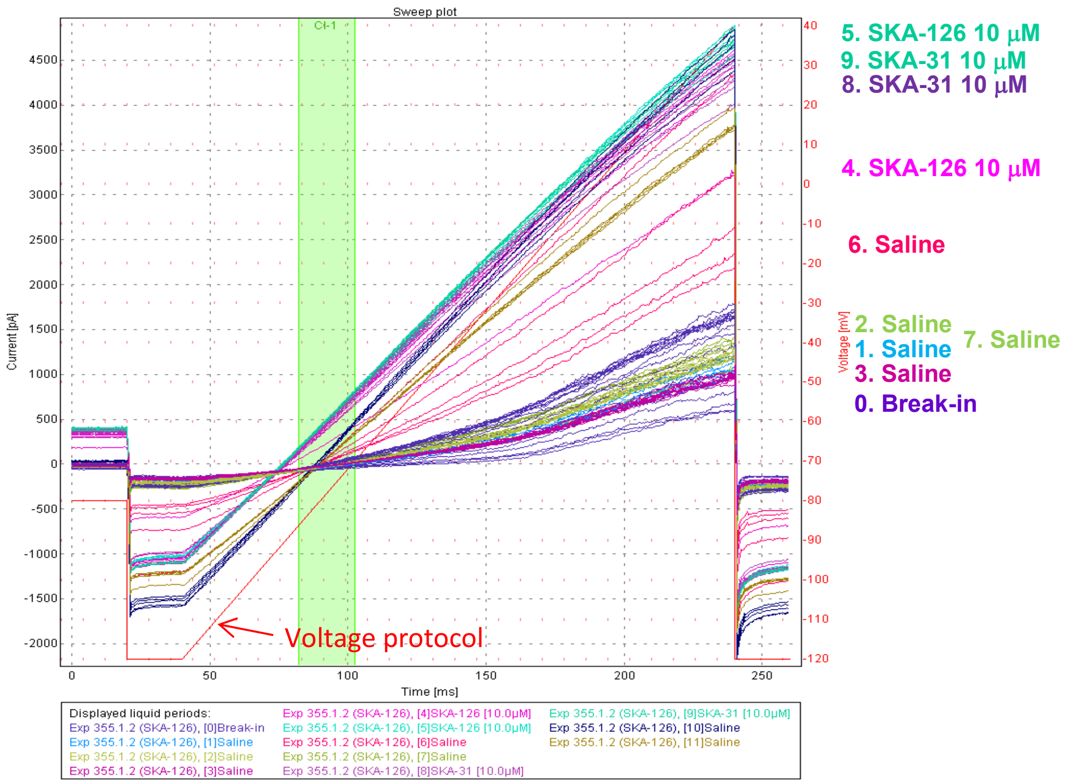

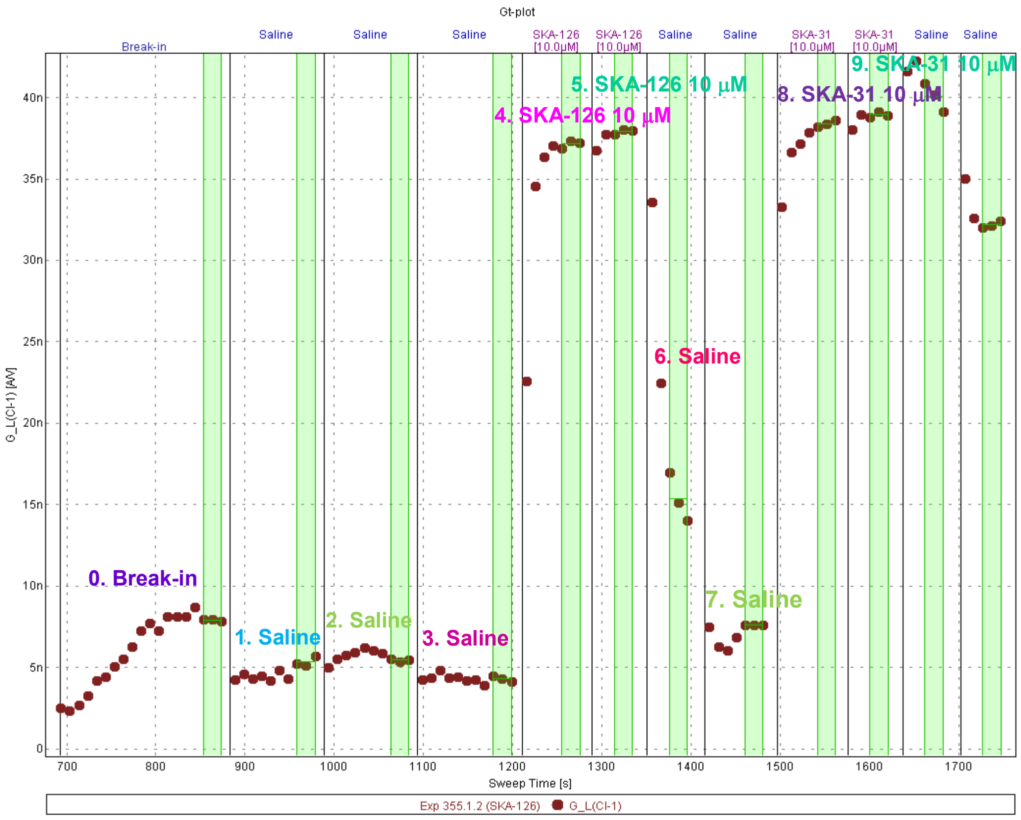

Raw current traces (top) and plot of current slope (measured between - 85 and - 65 mV) versus time for K_{Ca} activation (bottom). SKA-31 was used as the positive control. Saline or drugs were perfused during the first 15 sec of each so-called liquid period.

Screening of SKA-126 on $K_{Ca}2.2$

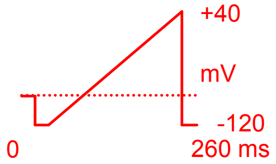

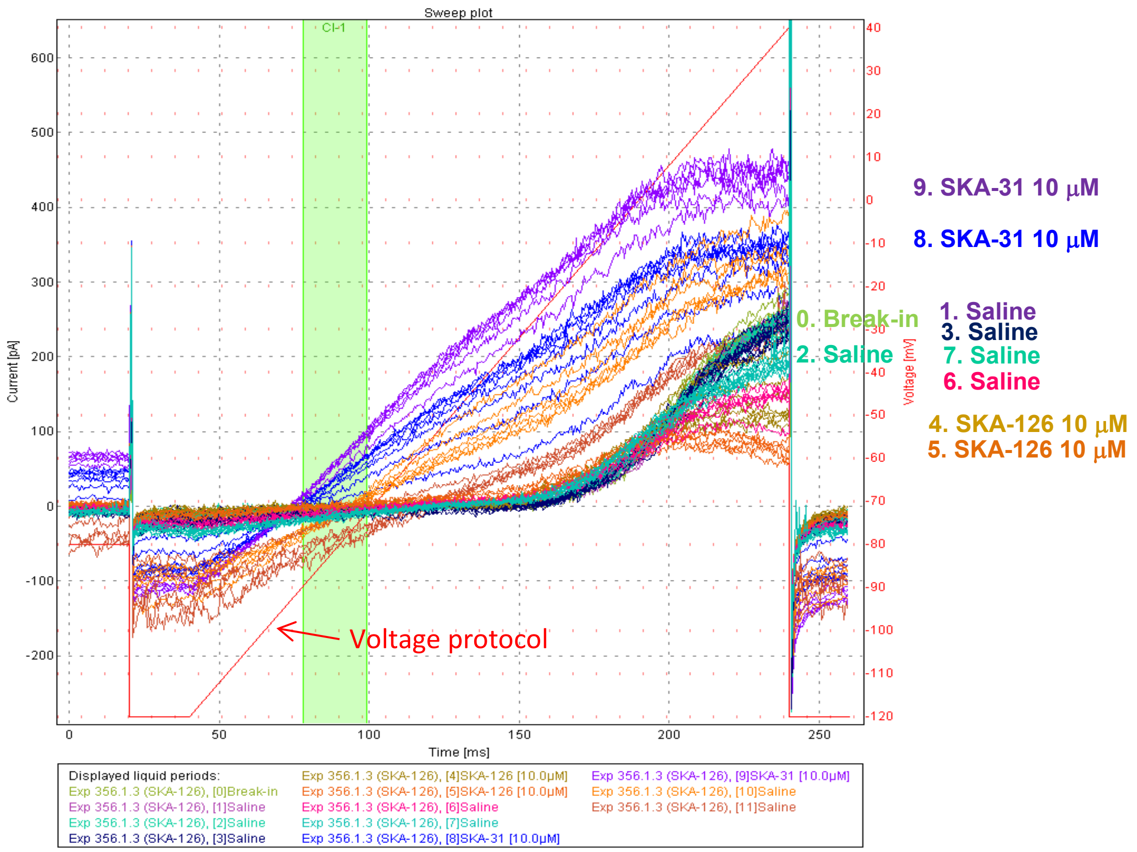

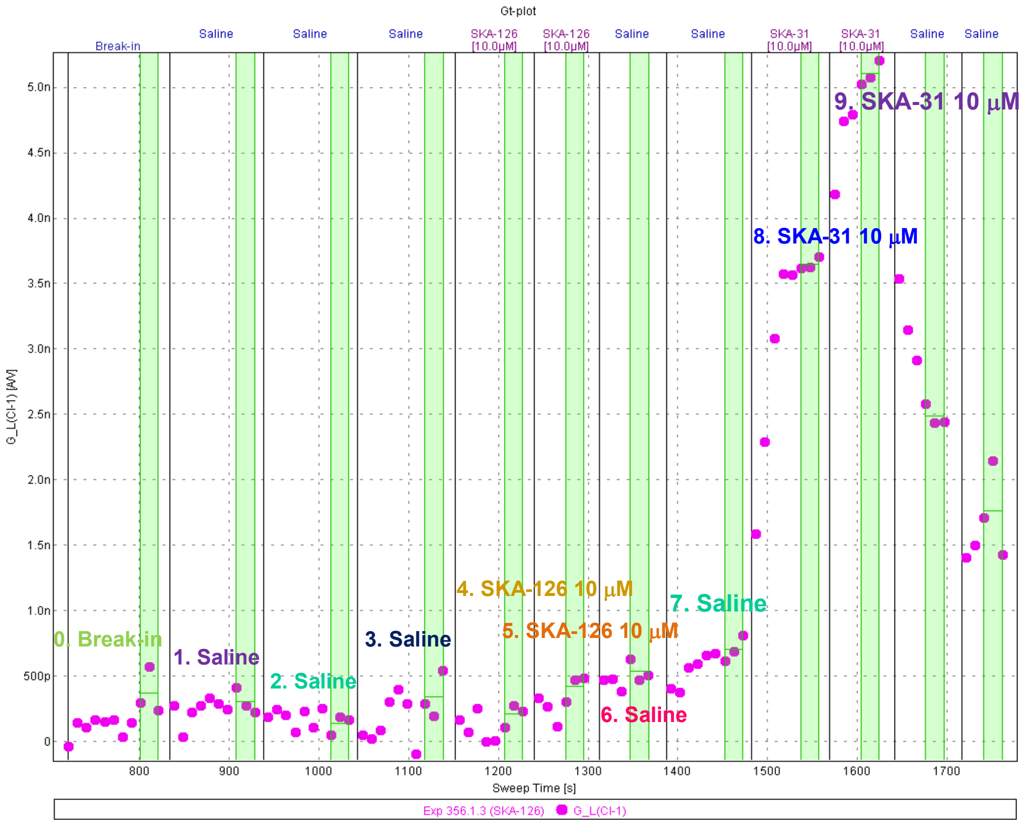

Raw current traces (top) and plot of current slope (measured between - 85 and - 65 mV) versus time for K_{Ca} activation (bottom). SKA-31 was used as the positive control.
Note: $K_{Ca}2.2$ currents are smaller than $K_{Ca}3.1$ currents and show some rectification at positive potentials.

Supplementary Fig.4

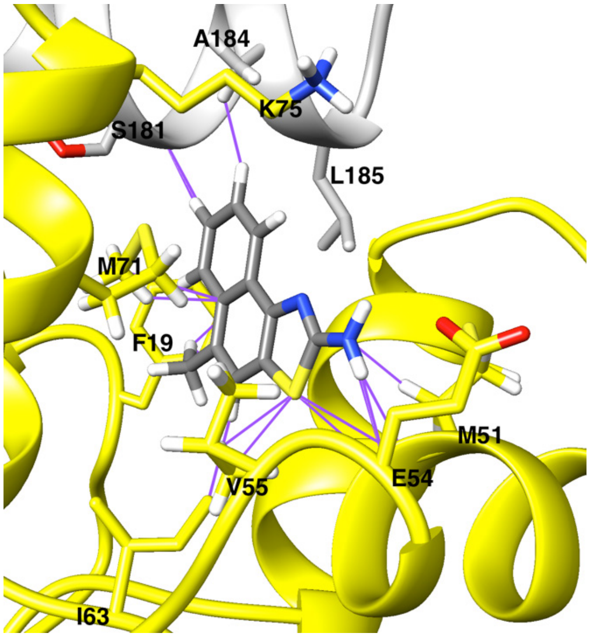

VDW interactions

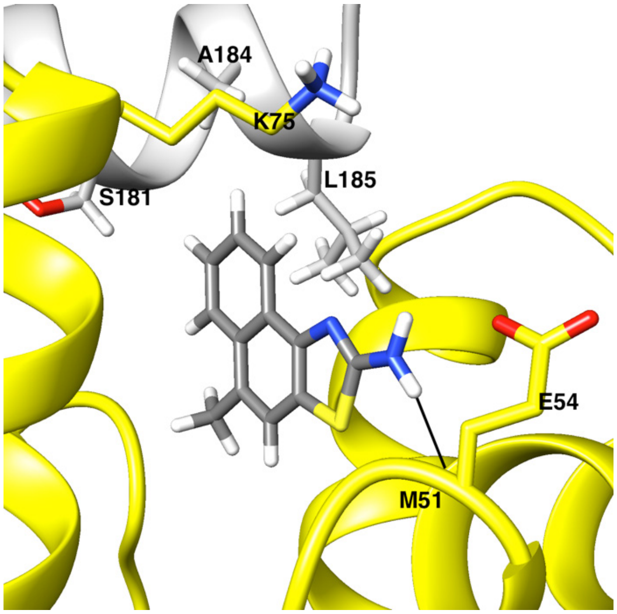

Hydrogen bonds

SKA-111 in K_{Ca}3.1 open state 2 (pdb id: 6cno)
The channel is shown in gray and the CaM N-lobe in yellow. Hydrogen bonds are shown in black, van der Waals interactions are visualized in purple. SKA-111 forms one hydrogen bond with M51 (right) and makes 12 van der Waals contacts with residues in the S_{45}A helix and the CaM N-lobe (left).

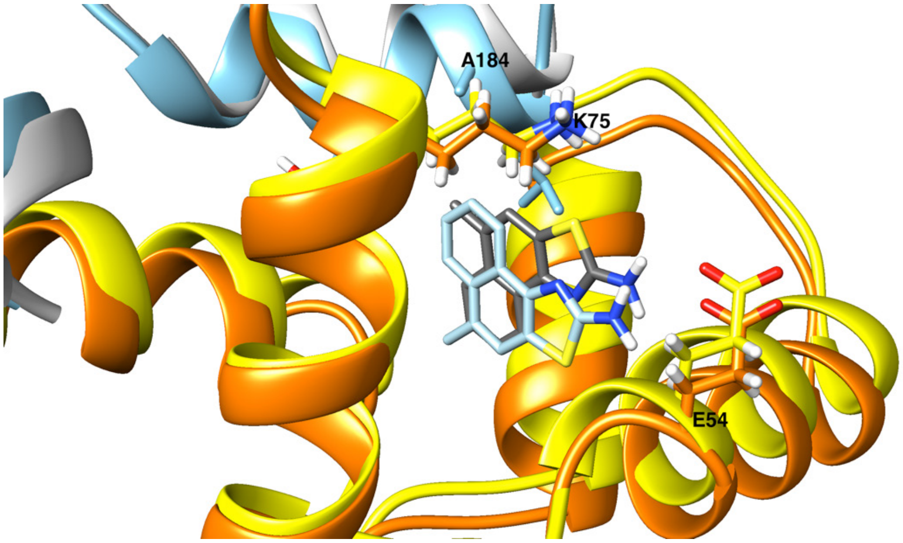

Overlay of the Rosetta docking models of SKA111 in open state 1 (yellow and light grey) and open state 2 (orange and sky blue). SKA-111 is shown in dark grey in open state 1 and in light blue in open state 2.

Supplementary Fig.5

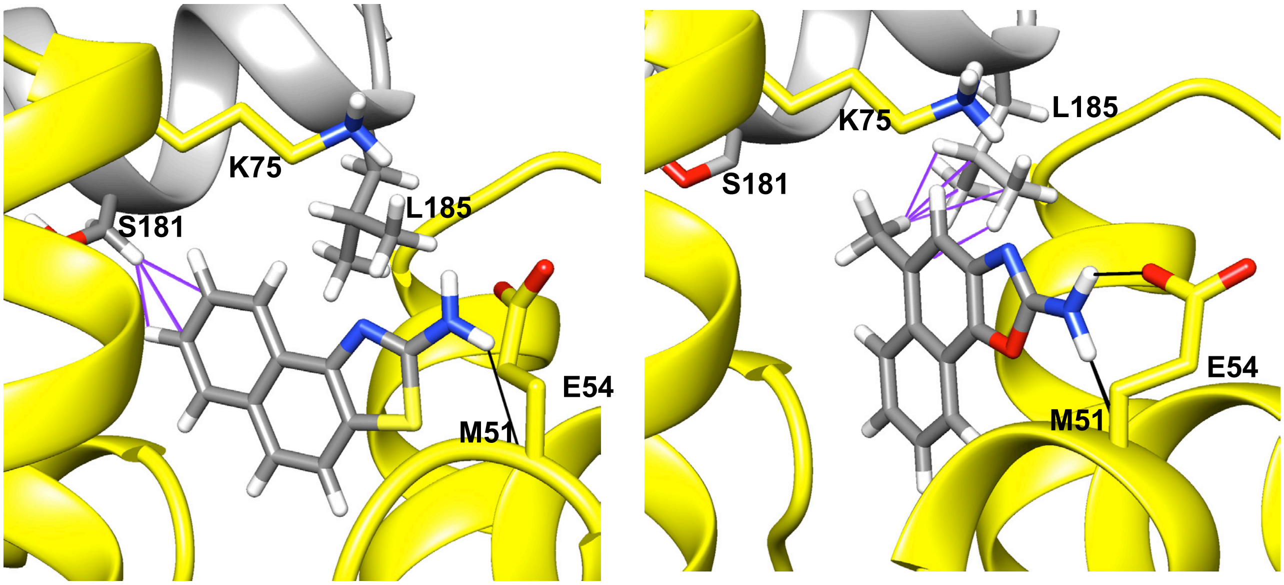

SKA-31 (left) and SKA-121 (right) in K_{Ca}3.1 open state 1 (pdb id: 6cnn)

The channel is shown in gray and the CaM N-lobe in yellow. Hydrogen bonds are shown in black, van der Waals interactions are visualized in purple. For clarity, not all side chains of CaM residues within contact range of ligand are explicitly shown.

Supplementary Fig.6

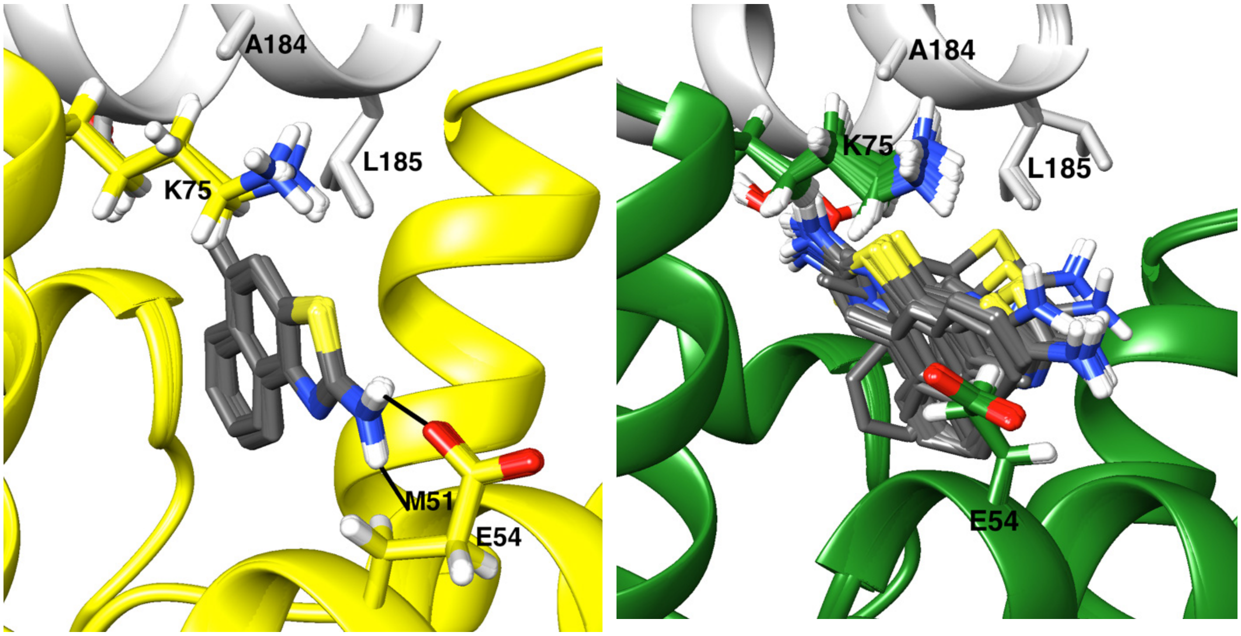

SKA-111 in WT **S372R**

Rosetta ligand docking models of the top 50 lowest energy-binding poses of SKA-111 in the interface between the CaM N-lobe (yellow: WT, green: S372R) and the S_{45}A helix (light gray) of wild type KCa3.1 and the S372R K_{Ca}3.1 mutant. Molecular docking shows that SKA-111 converged well in wild type K_{Ca}3.1 but not in the S372R KCa3.1 mutant channel.
